# Leukemic Stem Cell Frequency: A Strong Biomarker for Clinical Outcome in Acute Myeloid Leukemia

**DOI:** 10.1371/journal.pone.0107587

**Published:** 2014-09-22

**Authors:** Monique Terwijn, Wendelien Zeijlemaker, Angèle Kelder, Arjo P. Rutten, Alexander N. Snel, Willemijn J. Scholten, Thomas Pabst, Gregor Verhoef, Bob Löwenberg, Sonja Zweegman, Gert J. Ossenkoppele, Gerrit J. Schuurhuis

**Affiliations:** 1 Department of Hematology, VU University Medical Center, Amsterdam, The Netherlands; 2 Department of Medical Oncology, Inselspital, Bern University Hospital, University of Bern, Bern, Switzerland; 3 Department of Hematology, University Hospital Leuven, Leuven, Belgium; 4 Department of Hematology, Erasmus University Medical Center, Rotterdam, The Netherlands; Emory University, United States of America

## Abstract

**Introduction:**

Treatment failure in acute myeloid leukemia is probably caused by the presence of leukemia initiating cells, also referred to as leukemic stem cells, at diagnosis and their persistence after therapy. Specific identification of leukemia stem cells and their discrimination from normal hematopoietic stem cells would greatly contribute to risk stratification and could predict possible relapses.

**Results:**

For identification of leukemic stem cells, we developed flow cytometric methods using leukemic stem cell associated markers and newly-defined (light scatter) aberrancies. The nature of the putative leukemic stem cells and normal hematopoietic stem cells, present in the same patient's bone marrow, was demonstrated in eight patients by the presence or absence of molecular aberrancies and/or leukemic engraftment in NOD-SCID IL-2Rγ-/- mice. At diagnosis (n = 88), the frequency of the thus defined neoplastic part of CD34+CD38- putative stem cell compartment had a strong prognostic impact, while the neoplastic parts of the CD34+CD38+ and CD34- putative stem cell compartments had no prognostic impact at all. After different courses of therapy, higher percentages of neoplastic CD34+CD38- cells in complete remission strongly correlated with shorter patient survival (n = 91). Moreover, combining neoplastic CD34+CD38- frequencies with frequencies of minimal residual disease cells (n = 91), which reflect the total neoplastic burden, revealed four patient groups with different survival.

**Conclusion and Perspective:**

Discrimination between putative leukemia stem cells and normal hematopoietic stem cells in this large-scale study allowed to demonstrate the clinical importance of putative CD34+CD38- leukemia stem cells in AML. Moreover, it offers new opportunities for the development of therapies directed against leukemia stem cells, that would spare normal hematopoietic stem cells, and, moreover, enables *in vivo* and *ex vivo* screening for potential efficacy and toxicity of new therapies.

## Introduction

There is increasing evidence that the development of solid and hematological tumors depends on the presence of small populations of cells known as tumor-initiating cells or tumor stem cells [1]. The first proof of the stem cell concept came from studies by John Dick and colleagues in acute myeloid leukemia (AML) [2], [3]. In AML, these cells are referred to as leukemia-initiating cells or leukemic stem cells (LSCs) [4]. Since the first studies on LSCs, cell compartments defined by immunophenotype (CD34/CD38 expression) and function (side population, SP, and aldehyde dehydrogenase [ALDH] activity) have been reported to contain LSCs [5]–[9]. The first LSC compartment that was described had the CD34+CD38- immunophenotype [2], [3]. Although immune reactivity of the CD38 antibody used in earlier studies likely caused the lack of engraftment of CD38+ cells [10], the CD34+CD38- compartment still seemed to be the most robust compartment in CD34-positive (CD34+) patients, since it was found to be the predominant compartment containing leukemia-initiating cells in less immunocompromised mouse models [3]. On the other hand, in more severely immune-compromised mouse models, CD34+CD38+ and CD34-negative compartments were also found to contain leukemia initiating cells [5], [7]–[9], [11].

In bone marrow (BM) of AML patients, leukemic and normal cells are present within one compartment. The CD34+CD38- compartment in particular, was shown to contain both CD34+CD38- LSCs and normal hematopoietic stem cells (HSCs) [10], [12]. Previous papers reported only on the role of the size of the total CD34+CD38-stem cell compartment and only at diagnosis [13]–[17]. However, for proper identification of LSCs, with the aim to establish the prognostic impact of their frequencies at diagnosis and to follow their fate during and after therapy, it is important to identify distinguishing features between LSCs and HSCs. We previously have found that in a substantial number of AML cases, CD34+CD38- LSCs are characterized by the expression of C-type lectin-like molecule-1 (CLL-1) and aberrant expression of several lineage markers [18], [19]. Since these markers are absent on HSCs in regenerating BM after chemotherapy [18], [19], for the current study we chose to use these markers to distinguish between LSCs and HSCs in both diagnosis and post-diagnosis samples. Other LSC markers have also been described for AML diagnosis (reviewed in ref [20]), but since little is known about their behavior during and after therapy, their suitability for LSC tracking remains to be established. However, despite the usefulness of CLL-1 and lineage markers, in at least 25% of the AML patients, aberrant marker expression on CD34+/CD38- cells is absent or too weak, and therefore there is a need to identify other discriminative parameters [6], [19]. In the first part of this paper, we will describe such additional parameters for CD34+CD38- compartment. Now being able to discriminate neoplastic from normal CD34+CD38- cells, in the second part we will focus on the prognostic role of the CD34+CD38- stem cell compartment. This will be compared with the other CD34/CD38 defined compartments.

Moreover, another important aspect of cancer stem cells is their putative therapy resistance [21], with a subsequent ability to cause re-growth. The present study is the first to address the resistance to therapy by showing the prognostic impact of AML stem cells post-therapy in a large patient group. The results demonstrate that defining residual LSCs post-therapy has important prognostic impact. Moreover, we show that it adds important prognostic impact to a well-established immunophenotypical MRD approach, known to identify and quantify the bulk of neoplastic cells.

## Patients, Material and Methods

More detailed information can be found in the supporting text (Text S1).

### Patient treatment and sampling

Patients between 18 and 60 years of age with AML, except those with FAB M3 and previously untreated RAEB and RAEB-t patients (with IPSS ≥ 1.5), were included in this study. Detailed information regarding treatment can be found at http://www.hovon.nl. The HOVON/SAKK 42a and 92 studies were reviewed and approved by an institutional review board (METc) of the Erasmus MC Rotterdam for the total study (number 2000-220 for Hovon 42a) and 2008/216 for Hovon 92). In addition, the VU Amsterdam review board approved both studies with METc number 2001/50 (LUV) and 2008/292 (LUV), respectively. Patients provided their written informed consent to participate in this study. In 250 CD34-positive patients, we used a specific gating strategy to identify normal and neoplastic cells in the CD34+CD38-, CD34+CD38+ and the CD34- compartment at diagnosis. Patient details are in Table S1 (patient groups 1 and 2). Additionally, in the last part of the results, an extra patient group (n = 23) was included (details are in Table S1, patient group 3) to study combination of LSC and minimal residual disease (MRD) data after second cycle of therapy.

### Flow cytometry

#### Methods

LSC characterisation at diagnosis and LSC monitoring during follow up was performed on fresh patient samples. Purified white blood cells were obtained from BM or PB using lysing solution (Pharm lyse, Becton Dickinson, BD, San Jose, CA, U.S.A.) to eradicate red blood cells. After washing with PBS containing 0.1% human serum albumin (HSA), cells were re-suspended in PBS containing 0.1% HSA, incubated with monoclonal antibody combinations (mAbs) for 15 minutes at room temperature and washed with PBS containing 0.1% HSA. Details on antibodies (sources, clones, fluorochromes) are written in the supporting text (Text S1). Samples were analysed using a 4-color approach on a FACSCalibur from Becton Dickinson (BD, San Jose, CA, USA) using CellQuest and Infinicyte software. Cell sorting was performed using FACSAria (BD) with FACSDiva analysis software. More details are in the supporting text (Text S1).

#### Marker selection for the CD34+CD38- LSC

In a previous paper [19], we have shown that particular lineage markers, i.e. CD2, CD7, CD11b, CD19, CD22, CD56, can be positive on CD34+CD38- LSC, while always negative on CD34+CD38- HSC. This is true for CLL-1 too [18]. All markers have been shown to be negative on HSCs in normal bone marrow, but also on normal stem cells present at diagnosis and at follow up [18], [19]. In addition, the absence of CD13, CD33 and HLA-DR was considered aberrant, since these markers are always expressed on normal HSCs [19], [22]. For each new AML case one or more of these markers may be aberrantly expressed; in a next case this may be completely different. Therefore, for each new AML case, the choice for a marker was based on screening for presence (>10% positivity) of all markers. Even more heterogeneity was seen, since part of the CD34+CD38- population might be negative for these markers, and all or not covered by another marker.

#### Marker selection for the more mature CD34+CD38+ and CD34- populations

**Figure 1:**
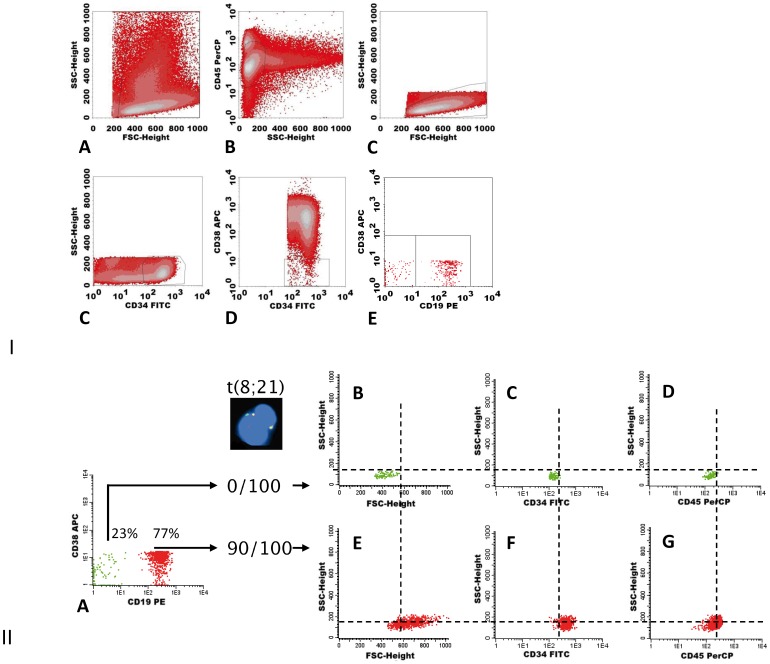
Gating strategy for the CD34+CD38- compartment and identification of pLSCs and HSCs in this compartment. I. Gating of CD34+CD38- AML cells. Cells were labeled with antibody-fluorochrome combinations as described in Patients, Materials and Methods. Remaining erythrocytes, debris and dead cells are largely excluded in an FSC/SSC plot (A). CD45^dim^/SSC^dim^ blast cells (B) were gated to homogeneity in FSC/SSC plot (C). CD34 positive cells are gated (D) and the CD38- stem cells are gated within this fraction (E). The CD38-negative fraction in D may contain two stem cell populations differing in CD34 expression (details in text). F. Within the CD34+CD38- gate, CD38 is plotted against an aberrant marker (in this case CD19) to indicate presence of putative LSCs (pLSCs) and HSCs. **II. Identification of pLSCs and HSCs.** This patient (nr 317) was diagnosed with t(8;21). Primary gating was as in I. Sorted CD34+CD38-/CD19+ cells (A, in red) were t(8;21) positive; sorted CD34+CD38-/CD19- cells (A, in green) were t(8;21) negative. These two populations were backgated in FSC/SSC (B,E), CD34/SSC (C,F) and CD45/SSC (D,G) plots. The CD19- cells are shown in the upper panels and the CD19+ cells in the lower panels. Dotted vertical lines (B–D) show that normal CD19- cells are FSC^low^ (B), CD34^low^ (C) and slightly lower in CD45 (D), compared to CD19+ cells (E–G). The dotted horizontal line shows that SSC of the normal stem cells (in green) was slightly lower than that of neoplastic stem cells (in red). FISH data are from an example published previously [19]. Similar results were found in an additional series of 7 patients (Tables 1 and 2). FSC and SSC of CD19+ pLSC were factor 1.71 and 1.77 higher than lymphocyte present in the same samples. FSC and SSC of the CD19 negative cells were only 1.08 and 1.20 times lower than lymphocytes.

For CD34+CD38+ and CD34- leukemic cells, identification had to be done using lineage markers: it is only lineage markers that are absent (or present at very low frequencies) on these more mature populations [23], [24]. This property offers the basis for detection of residual leukemic cells (all leukemic cells) in MRD approaches. CLL-1, however, cannot be used as a neoplastic marker on CD34+CD38+ and CD34- cells since it is present on part of these populations in normal bone marrow [25]. For LSC identification/quantification at diagnosis and follow up, only AML samples were used that, at diagnosis, showed>1% CD34 expression relative to the total WBC count. This was done because we have previously shown that the CD34+ population in AML with <1% CD34 is of normal origin [26]. CD34 negative AML samples, together with normal bone marrow, were used as a control for FSC/SSC values in HSC (see results section I.*A*). For details on the gating strategy to define the CD34+CD38- compartment, see Figure 1, part I. The details how to define scatter characteristics of marker positive and marker negative populations are outlined under Results. Stem cell numbers (LSC and HSC) were defined as a percentage of total white blood cells (WBC).

### Engrafting studies

Animal experiments were performed after approval of the animal ethical committee of the VU University, Amsterdam, The Netherlands under DEC-number: KNO06-02. NOD/SCID IL-2Rγ -/- mice were obtained from the Jackson laboratory (Bar Harbor, ME, USA). At the age of 8-10 weeks, the mice were irradiated sub-lethally with a dose of 350 cGy, 24 hours prior to transplantation of the human AML cells. Details of irradiation, anesthesia, intravenous and intra-femoral injection are outlined in the supporting text (Text S1) under “Patients”. Human leukemic and multi-lineage engraftment was determined based on positivity for CD45-PercP, the presence/absence of CD19-positive B-cells, CD13 and/or CD33-positive cells and, in three cases, by the presence/absence of aberrant marker expression.

Engraftment was defined as a clear clustered population in CD45 expression in a minimum of 200,000 acquired mice marrow cells. The minimum percentage of human engraftment that was detected was 0.1%, which shows up as a cluster of 200 cells on the scatterplot. Human leukemic engraftment was determined based on positivity for CD45-PercP, the absence of CD19-positive B cells and the presence of CD13 and/or CD33 positive cells: when CD13 was aberrantly absent on the AML that was injected, CD33 was used and when CD33 was absent, CD13 was used. Human multi-lineage engraftment was identified when CD45 positive cells consisted of both CD19 positive B-cells and myeloid cells with both CD13 and CD33 present (identified as monocytes and/or granulocytes in a FSC/SSC plot), and with absence of aberrant markers.

### FISH, FLT3-ITD and NPM1 analysis

For the FISH analysis, cytospins were prepared with FACS-sorted cells. LSI AML1/ETO dual color for t(8;21) probe (Vysis, Abbott molecular, Illinois, U.S.A.) was applied to the denatured cells and incubated as previously described [17]. Genomic DNA from sorted cell populations was analyzed for the presence of an FLT3-ITD as described before [27]. Mutations in NPM1 exon 12 were analyzed by PCR using genomic DNA that had been isolated from sorted cell fractions (see Text S1).

### Survival analysis

Statistical analysis of the stem cell data at follow-up was carried out using the SPSS 20.0 software program. The supplement provides details regarding the definition of overall survival (OS), event-free survival (EFS), relapse-free survival (RFS), Kaplan-Meier analyses, and Cox regression analyses for both univariate and multivariate analyses. P-values below 0.05 were considered significant.

## Results

In the absence of formal proof of leukemia initiating ability in the stem cell compartments of most of the samples studied, the different stem cell compartments are referred to as *putative* LSCs (abbreviated as pLSC).

Since the aim of the study was to define the prognostic impact of the neoplastic CD34+CD38- compartment, and to compare with the neoplastic CD34+CD38+ and CD34 negative compartments, first, properties will be described that allow to discriminate between the neoplastic and the normal compartment within the CD34+CD38- compartment (Section I.A–D) and the CD34+CD38+ and the CD34- compartments (Section I.E). Proof of the resulting concept was obtained using either molecular biological and/or murine engraftment experiments.

In Section II. the findings were used to assess the prognostic impact of the number of pLSCs at diagnosis and post-therapy for the CD34+CD38- compartment (II.A,B) and the CD34+CD38+ and CD34- compartment (II.C,D). Finally, the prognostic impact of the combination of follow up CD34+CD38- pLSC frequency, with MRD data will be described (II.E).

### I. Discrimination between leukemic and normal stem cell compartments

#### A. CD34+CD38-: discrimination of pLSCs and HSCs can be made based on aberrant marker expression and scatter properties

We have previously shown that the expression of aberrant markers on CD34+CD38- cells indicates the leukemic nature of stem cells [18], [19]. Figure 1.II illustrates the difference between CD34+CD38- pLSCs and HSCs based on aberrant marker expression and molecular aberrancies [in this example, t(8;21)]. In addition to Figure 1, Table 1 shows 7 other patients with aberrant marker positive cells, all with molecular aberrancies indicating that these are in fact neoplastic cells. The figure also shows that marker-positive pLSCs, compared to HSCs, are further characterized by a tight clustered cell population with higher forward scatter (FSC, reflecting cell size) and higher sideward scatter (SSC, reflecting granularity). This phenomenon was found in the other patients of Table 1 too (shown in Table 2). In Table 2, FSC and SSC of lymphocytes were used as internal controls to define the FSC/SSC position of pLSC and HSC. pLSC and HSC may also differ in CD34 and CD45 expression (in the case of Figure 1, CD34 expression of pLSC was higher than HSC). Such differences were not consistent but helped to define clusters of cells especially in cases with very low numbers of cells and/or small differences in aberrant marker expression and/or FSC/SSC.

**Table 1:** Molecular status of diagnosis total AML and sorted stem cell fractions from the same BM.

Patient nr	Molecular status of AML blasts§	Aberrant marker expressed onCD34+CD38- cells*	CD34+CD38- aberrant marker negative		CD34+CD38- aberrant marker positive	
			CD34+CD38-aberrant marker-	% aberrant marker- of CD34+CD38-$	CD34+CD38- aberrant marker+	% aberrant marker+ of CD34+CD38-
**317** ¥	t(8:21) pos	CD19	t(8;21) neg	23	t(8:21) pos	77
**808**	50% FLT3-ITD#	CD33-	wt	67	45% ITD#	33
	NPM1 mut	CD33-	wt	67	NPM1 mut	33
**945**	NPM1 mut	CLL-1	wt	47	NPM1 mut	53
**951**	42% FLT3-ITD	CLL-1	wt	46	60% ITD	54
**966**	43% FLT3-ITD	CD7	wt	41	41% ITD	59
**1263**	60% FLT3-ITD	CD33-	wt	2.5	80% ITD	97.5
**575**	50% FLT3-ITD	CD33-	wt	58	50% ITD	42
	NPM1 mut	CD33-	wt	58	NPM1 mut	42
**670**	NPM1 mut	CLL-1	wt	74	NPM1 mut	26

§ signal originating from the bulk of the leukemic blasts has been used for reference. Sorted lymphocytes from the same BM showed no molecular aberrancies.

^*^Aberrant markers with the highest coverage of the CD34+CD38- compartment were used for sorting HSC (aberrant marker negative) and pLSC (aberrant marker positive).

¥ Shown in Figure 1.

# For FLT3-ITD+ cases, the percentage of ITD of the total signal is indicated.

$ Median frequency of HSCs (46.5% of CD34+CD38- compartment) was higher than the median in a larger group of cases (median 18%), since cases in this table had to be selected on clear availability of both HSCs and pLSCs.

Abbreviations: wt, wild type: no FLT3-ITD or NPM1 peak present; mut: mutation of NPM1 (not quantitative), neg: negative, pos: positive, BM: bone marrow.

**Table 2:** FSC/SSC position relative to lymphocytes.

Patient nr	Aberrant marker negative CD34+CD38-	Aberrant marker positive CD34+CD38-
	FSC/SSC position*	FSC/SSC position*
**317** ¥	1.08/1.20	1.71/1.77
**808**	1.27/1.17	1.57/1.80
**945**	1.38/1.52	1.97/3.25
**951**	0.93/0.77	1.46/1.39
**966**	1.15/1.0	1.53/1.99
**1263**	1.04/1.22	1.39/2.08
**575**	1.06/1.02	1.34/1.77
**670**	1.21/1.07	1.52/1.73

¥ Shown in Figure 1.

* FSC/SSC values are based on the position of cells in FSC/SSC relative to the

FSC/SSC position of normal lymphocytes in the same BM sample.

The molecular status of the sorted cell fractions is summarized in Table 1.

Table S2 shows the FSC/SSC position relative to lymphocytes of CD34+CD38- HSCs present in normal BM, as well as CD34+CD38- present in CD34 negative AML, which have previously been shown to be normal [26]. FSC/SSC of HSCs present in these controls are all lower than pLSC present in CD34+ AML (Table S2). Moreover, HSC present in CD34+ AML also have low FSC/SSC and altogether there is not even overlap in FSC/SSC between pLSC in CD34+ AML and the HSCs present in these three bone marrow sources. This allows accurate definition of the nature of a population especially in those cases with only a single population present.

#### B. CD34+CD38-: is there a role of FSC/SSC to define pLSC and HSC in aberrant marker-negative part of CD34+CD38- compartment?

**Figure 2:**
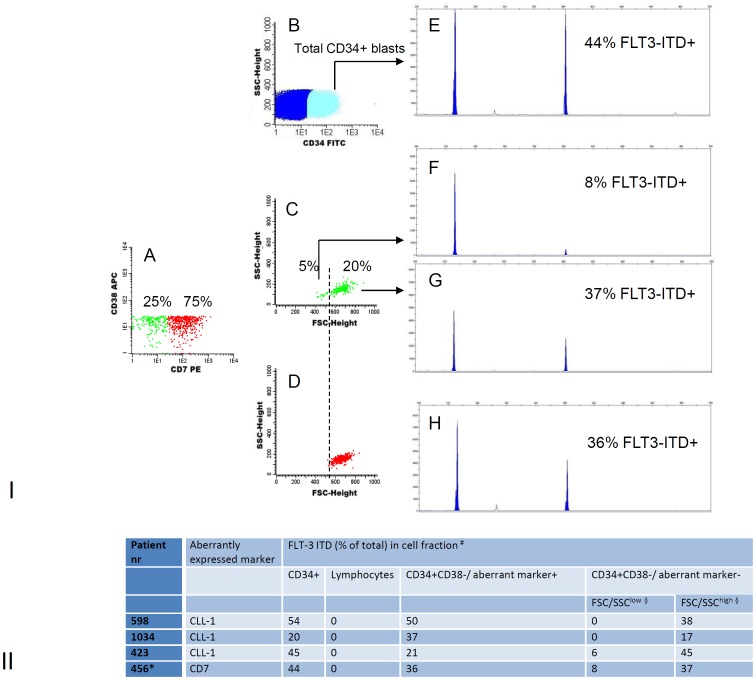
Marker-negative cells may contain leukemic cells defined by aberrant scatter. I. Gating/sorting strategy and molecular analysis for patient 456. CD34+ CD38- cells (patient 456, figure 2) were gated as in figure 1. CD34+CD38- cells were either CD7-negative (green in A and C, 25% of CD34+CD38-) or CD7 positive (red in A and D, 75% of CD34+CD38-). CD7+ cells were FSC/SSC^high^ (D) and of neoplastic origin (H). CD7-negative cells were further subdivided into FSC/SSC^low^ (left of the broken line in C), and FSC/SSC^high^ (right of the broken line in C). The CD7-negative, but FSC/SSC^high^, cells were neoplastic (G), while the CD7-negative FSC/SSC^low^ cells were essentially normal (F). CD34+ cells was the positive control (E). **II. Molecular analysis of stem cell subpopulations in four patients.** Sorting and analysis was done as in figure 2.I A for 3 additional patients. ^#^ FLT3-ITD (% of total signal: FLT3-ITD + wt) determined in cell populations sorted from CD34+ AML patients. * shown in figure 2.I A. ^§^ FSC/SSC^low^ and FSC/SSC^high^ defined as outlined in Table 2.

Aberrant markers may cover only part of neoplastic CD34+CD38- cells [19]. We therefore investigated whether scatter differences may help to discriminate pLSCs from HSCs in aberrant marker-negative cells. This was indeed seen as illustrated for patient sample 456 in Figure 2.I. The investigation of expression of only aberrant markers would have led to an over-estimation of HSC numbers and an under-estimation of pLSC numbers. Figure 2.II provides more examples.

**Figure 3:**
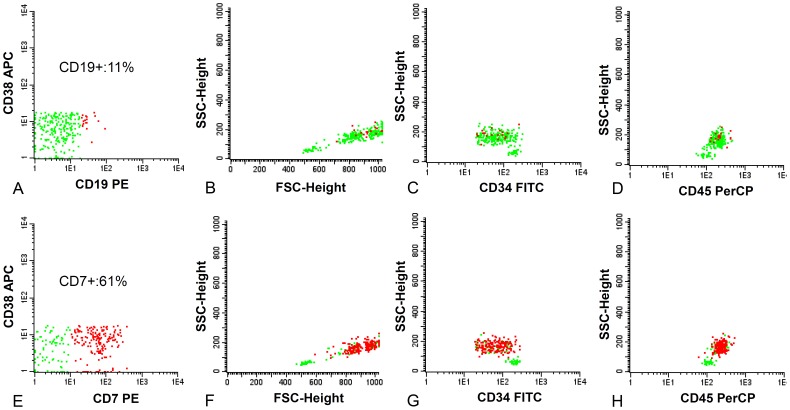
Marker negative pLSCs co-exist with marker positive pLSCs and are identified by scatter and CD34/CD45 expression patterns. CD34+ CD38- cells (patient 372) were identified as described in figure 1.I and gated for sub-compartments as described for figure 2.I. In the stem cell compartment of this AML case, only 11% could be identified as CD19+ (A). When back-gated in FSC/SSC (similar as performed in Figures 1 and 2), two different populations were identified based on the position in FSC/SSC: the small CD19+ fraction (events in red in A–D) is characterized by FSC/SSC^high^ (B), low CD34 expression (C) and high CD45 expression (D). CD19 negative cells (events in green in A–D), apart from a small FSC/SSC^low^/CD34^high^/CD45^low^ population of putative HSCs (A–D), contained a large population of cells that, similar to the CD19+ population in A–D, were FSC/SSC^high^ (B), CD34^low^ (C), and CD45^high^ (D). Apart from CD19, CD7 was an aberrant marker: 61% of the cells was CD7+ (E). Upon backgating, CD7+ cells (events in red in E–H), similar to CD19+ cells, were all FSC/SSC^high^ (F) CD34^low^ (G) and CD45^high^ (H). In contrast to CD19, CD7 negative cells (events in green in E–H) now completely consisted of a small FSC/SSC^low^ (F), CD34^high^(G), CD45^low^ (H) fraction. CD7 thus covered the whole neoplastic CD34+CD38- population and shows perfect discrimination between HSC and pLSC. CD19 expression in the absence of both CD7 expression and other scatter and CD34/CD45 expression parameters would have under-estimated the pLSC in the CD34+CD38- compartment by a factor 5.5 (61%/11%), while the HSCs would have been over-estimated by a factor 2.3 (89%/39%). In this case CD7 was a good marker to compare with the poor CD19 marker; it can be seen, however, that in the absence of CD7 expression, but with the scatter and CD34/CD45 aberrancies present, these would have enabled a complete discrimination between putative HSC and LSC compartments. This patient was identified as NPM1-positieve and FLT3-ITD positive. Other molecular aberrancies were not detected.

In approximately 25% of AML cases, there is no clear aberrant marker expression in the CD34+CD38- compartment, and in even more cases, marker expression is weak and overlaps with the marker negative cells [19]. In order to nevertheless discriminate between pLSCs and HSCs, we investigated whether scatter might replace marker expression in this respect. Figure 3 (A–D) shows that, even without clear aberrant marker expression (CD19 covers only a very low frequency CD34+CD38- population), differences in scatter can be used. Since, in this particular AML case, aberrant expression of CD7 was found (Figure 3 E–F), the validity of the scatter approach could be demonstrated (compare Figure 3 E–F with D). This shows that the scatter approach allows to discriminate pLSC and HSC in the absence of aberrant markers.

#### C. CD34+CD38-: discrimination between HSCs and pLSCs in a large patient group using marker and scatter differences

In cases with differences seen in FSC and SSC between pLSC and HSC, pLSC always had higher FSC/SSC than HSC. However, at this point it should be emphasized that differences in scatter were found in part of the patients. In 250 diagnosis AML cases studied, the combination of marker expression and FSC/SSC differences allowed accurate identification of both pLSCs and HSCs in 117/250 cases (47%), (outlined in detail in Table S3 and summarized in Table S4. For reasons mentioned earlier, differences in expression of CD34 and CD45 were only used for fine-tuning. As might be expected, the pLSC compartment made up the majority of the total CD34+CD38- compartment (median of 82%, ranging from 0%–100%). Apart from these 117 cases, there was an extra group of 102 patients (41%) with only marker expression available, while 31 patients (12%) had no marker and scatter properties usable for pLSC detection (summarized in Table S4).

#### D. CD34+CD38-: multilineage and leukemic engraftment of CD34+CD38- HSCs and CD34+CD38- pLSCs in NOD/SCID IL-2R γ^-/-^ mice

**Figure 4:**
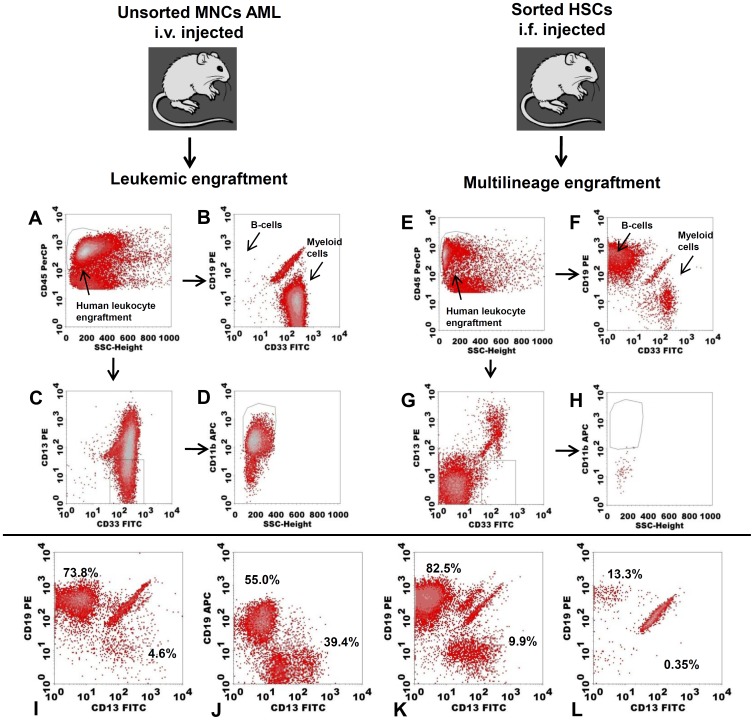
Multilineage engraftment of CD34/CD38 and scatter-defined putative HSCs. Unsorted mononuclear cells (MNCs) were injected intravenously and resulted in leukemic engraftment: cells were CD45+ (A) and of myeloid origin (B). In this case, the myeloid cells were positive for the diagnosis of leukemia-associated phenotype (LAP): partly CD33+CD13- (C) and CD11b+ (D). Sorted putative HSCs were injected intrafemorally (details, see Table S5). Engrafted CD45+ cells (E), contained both B-cells and myeloid cells (F), and lacked LAP (G,H). Multilineage engraftment of the sorted subpopulations was seen for patient 598 (I), 661 (J), 423 (K), and 928 (L). B-cells and myeloid cells (percentage of CD45+ cells) are in the upper left and lower right corners of the plots, respectively. The AML cells of patients 598 (I) and 661 (J) had an aberrant phenotype at diagnosis that was present in the neoplastic engrafted cells, but absent in the normal cells (not shown).

To provide further proof of principle for the strategy based on marker expression, secondary gating and molecular profiles, murine engraftment experiments were performed. HSCs were sorted by marker negativity, and clustering as FSC/SSC^low^, and/or distinct CD34/CD45 clustering, injected intrafemorally and evaluated for engraftment. In 5/6 cases, multilineage engraftment was found with no signs of leukemia (Figure 4, Table S5). In accordance with our *in vitro* gating strategy, AML engraftment with low cell numbers was observed in 2/6 cases for CD34+CD38- marker-positive samples and in 1/6 cases for the CD34+CD38- marker-negative cells, the latter being identified as neoplastic based on high FSC and aberrant CD34 expression (see legends of Table S5).

#### E. CD34+CD38+ and CD34-: discrimination between normal and leukemic CD34+CD38+ and CD34- cells and their leukemic engraftment in NOD/SCID IL-2R γ^-/-^ mice

Although emphasis in this paper is on the CD34+CD38- compartment, results described later for prognosis of CD34+CD38- pLSC will be compared with the other CD34/CD38 defined compartments. To enable such, neoplastic CD34+CD38+ and CD34- cells were identified by aberrant expression of markers used to define so-called Leukemia Associated (Immuno) Phenotypes established at diagnosis and used by others and us [23], [24] for detection of minimal residual disease. Cells were sorted as described in Text S1. In agreement with recent reports, these cell compartments also had leukemia initiating potency since AML engraftment was found in 4/6 cases for the CD34+CD38+ compartment and in 2/6 cases using CD34- compartments (referred to in the legends of Table S5). In all but one case, this was accomplished with high cell numbers (100,000-1,000,000).

### II. Prognostic role of putative stem cell compartments

To assess the clinical impact of our findings, we assessed the prognostic value of the size the three individual LSC compartments both at diagnosis and, where applicable, especially at clinical follow-up.

#### A. CD34+CD38- at diagnosis

**Figure 5:**
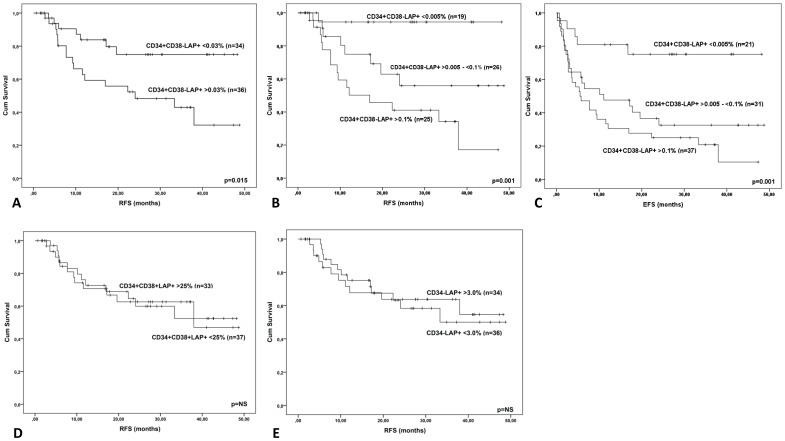
Prognostic value of frequencies of pLSC compartments at diagnosis. This figure shows the Kaplan-Meier analyses at diagnosis for the three compartments putatively containing pLSCs: CD34+CD38- (A,B,C), CD34+CD38+ (D) and CD34- (E). Of the 117 patients shown in Table S3, for Figures 5 and 6, 88 patients were chosen who had at least one follow-up time point. Of these, 70 entered Complete Remission, of whom 53 after the first course, 13 after the second course and 4 at later stages. Eighteen never reached CR. The size (median values) of the CD34+CD38- compartment at diagnosis was significantly (six-fold) higher in patients who did not enter CR (n = 18) compared with patients who did (n = 70): 0.225% of WBC versus 0.036% of WBC (p = 0.041). For CD34+CD38+ and CD34-, there were no significant differences (see text). Cut-off levels were defined to divide the total population into high stem cell frequencies (above cut-off) and low stem cell frequencies (below cut-off). A particular cut-off value was chosen (A, D, E) to ensure approximately equally numbers of patients in the resulting high and low stem cell frequency compartments. Results for other cut-offs for the three pLSC compartments are in Table S6. A–C: CD34+CD38-; D: CD34+CD38+; E: CD34-. A. RFS in remission patients (n = 70) with diagnosis CD34+CD38- cut-off of 0.03%; B. RFS in the same patient group (n = 70), but now with 2 cut-offs (0.005% and 0.1%); C. Event-free survival for all CR and non-CR patients (n = 88); D. RFS in remission patients (n = 70) with CD34+CD38+ cut-off of 25%; E. RFS in remission patients (n = 70) with CD34- cut-off of 3%. All relevant prognostic variables with statistical significance were investigated in a multivariate model. In this multivariate analysis it was found that risk group (according to the HOVON 102 trial) was an independent prognostic factor for OS at diagnosis (p = 0.001). For RFS, both risk group and CD34+CD38- leukemic stem cell load (using a 0.03% cut-off point) were independent prognostic factors at diagnosis (p = 0.017 and p = 0.011, respectively).

To assess prognostic impact, different cut-off values were established to define patients with high pLSC count (above the cut-off level: referred to as pLSC+) and patients with low pLSC count (below the cut-off level: pLSC-). Patients who later turned out not to have achieved complete remission (non-CR patients) had median 6-fold higher pLSC count than patients who had achieved CR (see legends of Figure 5). Moreover, the CR patient group could be divided into two groups with significantly different prognoses at multiple cut-off points ranging from 0.002% to 1% (Figure 5A and Table S6A). Figure 5B shows that further splitting up the CR group resulted in the definition of three groups with large differences in relapse-free survival (RFS). The latter result was also found upon inclusion of non-CR patients (not shown) and replacement of RFS by EFS (Figure 5C). Multivariate analysis showed pLSC frequency to be an independent prognostic factor (Figure 5, legends).

#### B. CD34+CD38- during follow-up in CR

**Figure 6:**
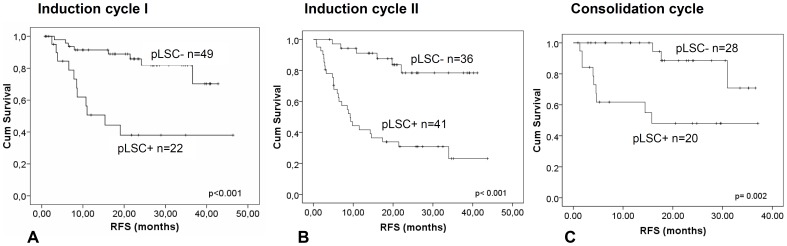
Prognostic value of frequencies of CD34+CD38- pLSC compartment at follow-up. This figure shows the Kaplan-Meier analyses for RFS for the CD34+CD38- pLSC compartment at follow up for three consecutive therapy cycles. The optimal cut-off levels were chosen to define pLSC + and pLSC- after 1^st^ induction cycle (0.0003%,which is 3 pLSCs in 1,000,000 WBC) and after 2^nd^ induction cycle and consolidation therapy 0.0001% (1 pLSC in 1,000,000 WBC). Results for other cut-offs are in Table S7. After the first induction cycle (B, 71 patients), second induction cycle (C, 77 patients), and after consolidation therapy (D, 48 patients), patients with high pLSC frequency (pLSC+) showed significantly more adverse performance compared with patients with low pLSC frequency (LSC-).

The gating approach shown in Figure 1 was used to follow the fate of pLSCs over time using sequential sampling (examples in Figure S1). Cox regression analysis showed a strong significant inverse correlation between the pLSC percentage after all therapy cycles and RFS and OS (for details, see legends to Table S7). Kaplan-Meier analyses showed markedly improved RFS and OS in pLSC- patients compared with pLSC+ patients (example in Figure 6, A–C), which applies for a large range of cut-off values (Table S7). Multivariate analyses with cut-off values showed that pLSC frequency after the first and second treatment cycles was an independent prognostic factor for RFS and OS (example with 0.0003% and 0.0001% shown in Table S8).

#### C. CD34+CD38+ and CD34- at diagnosis and at follow up

The CD34+CD38+ and CD34- compartments have been shown to contain leukemia initiating cells [5], [11] (also in this paper). In order to assess whether these were clinically important, when present together with the leukemia initiating CD34+CD38- compartment, we assessed their prognostic impact.

*At diagnosis,* neoplastic CD34+CD38+ and CD34- blast cells were identified as described under I.B. For CD34+CD38+, there was no single cut-off value (tested in the range 2%–60%) that resulted in discrimination of two patient groups with different RFS (Figure 5D; Table S6B), even when non-CR patients were included in the latter (data not shown). Although only borderline significant, in contrast to CD34+CD38-, the neoplastic CD34+CD38+ compartment was even smaller in non-CR patients than in CR patients (10.8% of WBC versus 23.7% of WBC; p = 0.06). Similar results were obtained for the CD34- compartment with cut-off points ranging from 0.1%–30% (Figure 5E; Table S6C), with the neoplastic compartment size, similar to CD34+CD38+, being even smaller (albeit non-significantly) in non-CR patients than in CR patients (0.87% of WBC versus 2.41% of WBC, p = 0.48).

*At follow up*, the neoplastic component of the CD34+CD38+ and CD34- compartment represented a considerable portion of the total neoplastic blast compartment. This reflects the total leukemic burden, or MRD, which, in turn, has previously been shown by many authors, including ourselves, to have prognostic impact (see also next paragraph) [23], [24].

#### D. Post-diagnosis prognostic impact of CD34+CD38- LSC combined with MRD

**Figure 7:**
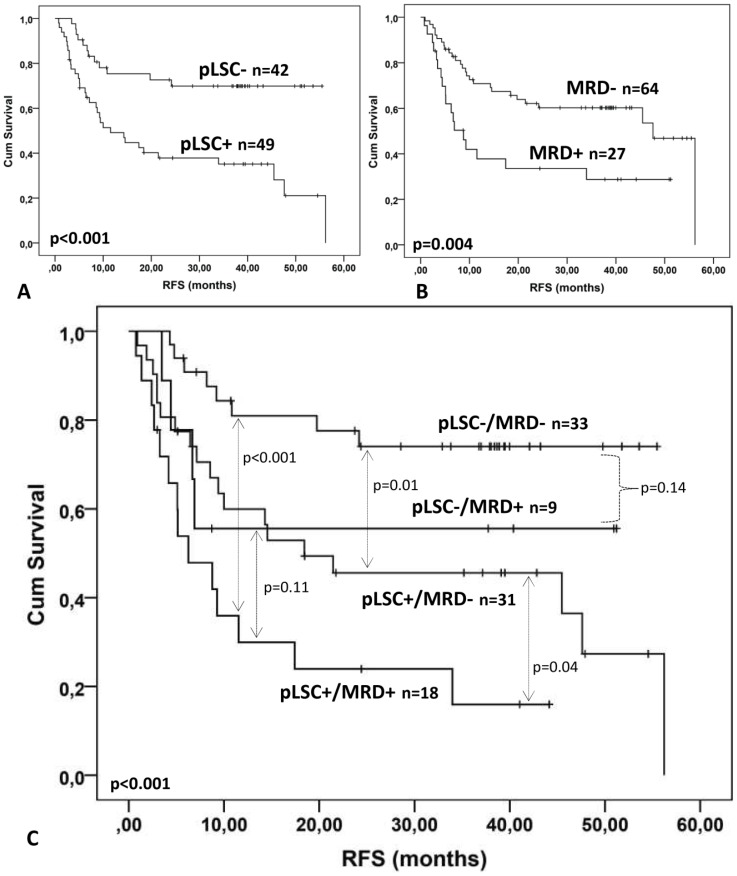
Prognostic value of combined p-LSC and MRD. (A) Kaplan-Meier analyses after cycle II for RFS for the pLSC data as shown in Figure 6, with an additional 23 patients (Table S1). The pLSC cut-off used is 0.0001%. (B) Kaplan Meier analysis of MRD data (cut-off 0.1%) obtained for the same patient group as in A (n = 91). (C) Combined pLSC and MRD (n = 91) data resulted in 4 patient groups: pLSC-/MRD-, pLSC-/MRD+, pLSC+/MRD- and pLSC+/MRD+.

The burden of leukemic stem cells after chemotherapy does not always reflect the total leukemic burden (known as MRD). It can thus be argued that the combination of pLSC frequency and MRD frequency may improve prognostic information at follow up. Because this combination would divide the total patient group in four relatively small sub-groups defined by both a pLSC cut-off (Figure 6, Table 3) and a fixed MRD cut-off of 0.1 [24]), we increased the size of the present patient remission group by including 23 similarly treated young adults in remission (<65 years) (details in Table S1 “Patients 3”). In the total group (n = 91) pLSC frequency again had strong prognostic impact over a range of cut-off points (from 0 in 10^6^ WBC up to 10 in 10^6^ WBC. As an example, the cut-off point of 0.0001% is shown in Figure 7A. For MRD, the cut-off point of 0.1% had the expected prognostic impact (Figure 7B) [24]. When combining MRD and pLSC in Figure 7C, four groups were identified. The main conclusions from this figure are: 1) within the total MRD- group (n = 64), pLSC+ patients (n = 31) have significantly poorer prognosis than pLSC- patients (n = 33; p = 0.01); 2) within the pLSC+ group, MRD- patients, although having a relatively poor prognosis, may do better than MRD+ patients (p = 0.04); the pLSC-/MRD- group had relatively good prognosis, while the pLSC+/MRD+ group had very poor prognosis.

**Table 3:** Role of cytogenetically/molecularly defined risk groups in pLSC/MRD defined sub-groups.

Group	N (nr per pLSC/MRD sub-group)	% per pLSC/MRD sub-group	40 months survival* (%)
sub-group (cytogen/mol)			
**pLSC-/MRD- (total)**	**33**	**100**	**74**
**good**	8	24	69
**intermediate**	11	33	80
**poor**	10	30	80
**very poor**	4	12	50
**pLSC-/MRD+ (total)**	**9**	**100**	**56**
**good**	5	56	60
**intermediate**	3	33	67
**poor**	0	0	-
**very poor**	1	11	0
**pLSC+/MRD- (total)**	**31**	**100**	**45**
**good**	8	26	47
**intermediate**	7	23	57
**poor**	12	39	47
**very poor**	4	13	0
**pLSC+/MRD+ (total)**	**18**	**100**	**16**
**good**	4	22	25
**Intermediate**	2	11	0
**Poor**	7	39	15
**Very poor**	5	28	0

* 40 months was chosen as most survival curves had reached a plateau.

MRD cut-off: 0.1% of WBC, pLSC cut-off: 0.0001% of WBC.

When including the distribution of cytogenetically/molecularly good, intermediate, poor and very poor patients, these were all represented in the four LSC/MRD defined subgroups (Table 3). This shows that, the pLSC/MRD prognostic impact is across cytogenetic risk groups, although in the LSC+/MRD+ group poor and very poor cytogenetic/molecular risk groups are prevalent.

When including the post-diagnosis prognostic parameter “cycle after which CR is reached” in Figure 7, it turned out that there was no significant difference between the first three pLSC/MRD defined patient groups in number of cycles needed to reach CR: in the first group (pLSC-/MRD-) 29 after first cycle versus 4 after second cycle; in the third group (pLSC+/MRD-) this was 25 versus 6. The second group (pLSC-/MRD+) was too small (all patients in CR after one cycle). However, in the fourth group (pLSC+/MRD+) for 9 patients two cycles were needed, with one for the other 9 patients.

These data show that combining cytogenetic/molecular defined risk groups together and clinical parameters like cycles to CR, together with pLSC/MRD defined risk assessment may offer a very important new algorithm in risk assessment.

## Discussion

One of the major challenges in the design of new therapies to eradicate leukemia stem cells is to achieve high therapeutic specificity. To this end, it is important to distinguish LSCs from the concomitantly present HSCs, and to assess whether this distinction is of prognostic value, since it would underline the clinical importance of LSCs. Consequently, it was necessary to identify parameters that allowed discrimination between these pLSCs and HSCs, preferably in all AML cases. Recent studies have shown that pLSCs may reside not only in CD34+CD38-, but also in CD34+CD38+ and CD34- compartments [5], [11]. In the present paper, we first present methods to discriminate between the neoplastic and normal portions of these compartments, and we subsequently assessed the prognostic value of these putative stem cell compartments.

With regard to the prognostic impact, using a uniquely-designed multi-parameter flow cytometry protocol, we show for the first time that the CD34+CD38- pLSC load after different cycles of therapy was highly predictive of patient survival, independent of other prognostic parameters. In addition, following our own preliminary studies [13], we identified three patient groups at diagnosis defined by CD34+CD38- pLSC with very large differences in prognosis, again independent of other prognostic parameters. By comparison with literature [13]–[15], it can be appreciated that the prognostic impact using our new approach, in which the pLSC compartment within the total CD34+CD38- compartment was specifically used, is much higher than using the total CD34+CD38- compartment as done in the previous studies. This is likely due to the “contamination” of the leukemic CD34+CD38- compartment with HSCs in the earlier studies (ranges of 0%–100% of pLSC, as seen in our study, also means that HSC range from 0%–100%), as seen in Table S3. In contrast, the CD34+CD38+ and CD34- compartments at diagnosis completely lacked prognostic impact, which strongly suggests that CD34+CD38+ and CD34- pLSCs are of minor clinical importance, at least in AML cases where these compartments are accompanied by CD34+CD38- pLSCs (as by definition is the case in our current CD34 positive patient group). However, at follow-up, leukemic CD34+CD38+ and CD34- compartments represent considerable portions of the total leukemic burden and thus reflect MRD cell frequency rather than pLSC frequency. Here, CD34+CD38+ and CD34- cells probably originate from (limited) differentiation of the CD34+CD38- pLSCs, a process that has been shown to occur *in vivo* by Goardon and colleagues [28].

The results are compatible with the following model: what the paper shows is that in CD34 positive AML cases, it is the percentage of the CD34+CD38- population at diagnosis that strongly correlates with clinical outcome and not the percentage of CD34+CD38+ or CD34- cells. This does not mean that CD34+CD38+ and CD34- cells do not contain leukemia initiating ability; it simply strongly suggests that, in the presence of CD34+CD38- cells, these CD34+CD38+ and CD34- leukemia initiating cells are either less therapy resistant and/or less malignant compared to CD34+CD38- cells. Likely, leukemia initiating ability in mouse models of CD34/CD38 defined sub-populations do not reflect clinical importance (see also next paragraph), since this ability is always assessed using purified populations, whereby the “competition” between these populations in outgrow and/or the relative therapy resistance cannot be taken into account. This model also implies that in CD34 negative AML (with only neoplastic CD34- populations present), it is the CD34- pLSC that takes over the leukemia initiating ability. Also in CD34 positive AML in the absence of CD34+CD38- cells, but with neoplastic CD34+CD38+ and CD34- populations present, the latter two populations may take over the leukemia initiating ability. As a logical consequence of the model these putatively less aggressive and/or less therapy resistant populations should define a better clinical outcome, which is indeed the case: in a separate cohort of 438 patients, survival of CD34 negative patients was significantly better than survival of CD34 positive patients (unpublished results).

The lack of correlation between prognosis and the size of the different CD34+CD38+ and CD34- compartments, while all compartments in purified form do engraft in a mouse model, may be explained as follows: the overall immune status of a patient group like that in our study, may best be represented by a less immune-restricted mouse model, where CD34+CD38- pLSCs are the predominant engrafting cells [29], [30]. In line with that, in our earlier engrafting experiments using CD34+ cells (i.e., containing CD34+CD38-, CD34+CD38+ and CD34- cells) in the less immune-restricted NOD/SCID mice, it was only the size of the CD34+CD38- compartment at diagnosis that correlated with levels of engraftment [13]. More immune-restricted mouse models are useful to study LSC engraftment of probably less aggressive pLSC sub-populations [5], [28]. In this respect, an important initial observation was made by Costello and co-workers, who found that, *in vitro*, CD34+CD38- cells were more therapy-resistant and less immunogenic than other compartments [21]. Moreover, further compelling evidence that the CD34+CD38- compartment is most important in the clinical setting comes from our clinical observations: in most cases which had low frequency mutations at diagnosis, which became predominant at relapse, these mutations were present and/or enriched in the neoplastic CD34+CD38- diagnosis compartment [31].

Survival and outgrowth of leukemia cells after therapy may depend on many factors and include the LSC load and likely specific LIC properties, but also the frequency of AML blast cells referred to as MRD. Many authors, including us, have shown that MRD is a strong independent prognostic factor [24], [32], [33]. In this paper we have shown that both CD34+CD38- pLSC frequency and MRD cell frequency are complementary, thereby defining a new post-diagnosis combination factor that offers strong prognostic information, even across cytogenetics/molecular defined risk groups. This will prospectively be validated in a new patient cohort of the HOVON/SAKK cooperative study group for which patient enrollment has already started.

With regard to our approach to distinguish between pLSCs and HSCs within the CD34+CD38- compartment, we have shown that aberrant expression of antigens (including CLL-1) and lineage markers, as well as additional aberrancies (including differences in CD34 and CD45 expression and differences in light scatter) allow unequivocal discrimination between pLSCs and HSCs. Moreover, this approach even allowed CD34+CD38- pLSC detection in patients lacking aberrant antigen expression. By applying the additional parameters, underestimation of pLSC numbers or overestimation of HSC numbers (often seen when using marker expression alone) is avoided. The present study thereby confirms the findings of others [29] that different CD34+CD38- pLSC markers expressed in individual AML cases may miss substantial portions of LSCs present (Figure 3). Although secondary parameters are thus very useful, it requires experience.

The number of accurately-identified pLSCs would increase with the use of additional AML markers. Such markers may include: CD123, CD96, CD44, CD47, CD25, CD32, CD33, TIM-3 [12], [18], [29], [34]–[38]. In the present study, both lineage markers and CLL-1 were used to identify CD34+CD38- pLSCs and HSCs during follow-up, since these markers remained highly specific for these LSCs during and after therapy [18], [19]. To date, other markers have not yet been tested and considerable efforts will be necessary to demonstrate their applicability both for pLSC quantification at diagnosis and especially at follow up, and as putative targets in antibody therapies. In particular, undesirable post-chemotherapy up-regulation on HSCs may occur for some of these markers [18], [19]. Thus, our study implies that, for adequate pLSC (and HSC) tracking, a spectrum of markers and probably additional parameters should be screened for each individual AML case. Therefore, broad application of a single target antigen for diagnostic purposes, as well as for future LSC-directed antibody therapies, still seems unlikely.

Although FSC/SSC characteristics are often effective in discriminating between CD34+CD38- pLSCs and HSCs, the underlying cause of this difference is not yet known. An attractive option is that, similar to normal stem cells, CD34+CD38- AML sub-populations may exist with slightly different levels of differentiation [28]. In fact, we now have more formal proof for that: AML cases with high FSC and SSC of the CD34+CD38- cells all have expression of the differentiation marker CD45RA. It is known that this marker identifies GMP CD34+CD38+ progenitors in contrast to MEP and CMP CD34+CD38+ progenitors. We have found that CD45RA is completely absent on the real CD34+CD38- HSCs, but marked corresponding CD34+CD38- pLSC populations in roughly half of the AML cases. In the other half, the pLSC had FSC and SSC close to those of corresponding HSCs; these pLSCs were CD45RA negative. The CD45RA positive cases may indeed reflect more progenitor-like pLSCs compared to the CD45RA negative pLSCs that resemble HSCs [28].

In AML cases with no malignant CD34+CD38- compartments, the pLSCs will be located in the CD34+CD38+ and/or CD34- compartments. It is likely, however, that within these relatively large compartments, further compartmentalization will be necessary to identify the true LSC sub-compartment which likely occurs at low frequencies. One candidate fraction is the side population (SP), which does occur in low numbers (usually <1% of the WBC population) and contains both normal and AML cells at diagnosis [8], [9]. Moreover, the SP has also been found to contain different CD34/CD38-defined sub-fractions [6], which may represent CD34/CD38/SP pLSCs. In the future, it would be interesting to examine the relationships between the functional phenotypes (based on SP or aldehyde dehydrogenase activity) [6]–[9], [39], [40] and the CD34/CD38 immunophenotype.

In conclusion, the present study offers tools for detecting concomitantly present pLSCs and HSCs, both at diagnosis and at disease follow-up, and provides the first proof that CD34+CD38- pLSCs are not only clinically important at diagnosis, but also at follow-up. No evidence was found to suggest that the CD34+CD38+ and CD34- leukemic fractions contain clinically important LSCs, at least not in CD34 positive AML with leukemic CD34+CD38- present. The combination with well-established MRD assessment likely opens a new field in prognostication in patients with AML. Ultimately, our findings may contribute to the development of new diagnostic tools and to novel, more selective therapies, including antibody-based therapies that would be highly effective against AML stem cells, while leaving the normal HSCs intact. Finally, these results may stimulate further research into the role of cancer stem cells in other cancers, such as solid tumors.

## Supporting Information

Figure S1 **pLSCs monitoring during sequential BM sampling.** A patient (243) with AML positive for a t(8;21) showed CLL-1 expression in the CD34+CD38- compartment (A), while normal BM misses CLL-1+ CD34+CD38- cells [1]. In CR, CLL-1+ CD34+CD38- cells (B) were sorted and assessed for t(8;21): mainly neoplastic cells were present (E). Normal CD34+CD38-CLL-1 negative cells were almost completely absent here. Shown in F-H are examples of sequential monitoring in three cases with increasing periods of complete remission until relapse (F, pt 253; G, pt 372) and in continuous remission (H, pt 572). Note the increase of pLSC frequency preceding relapse (F, G).
(PPTX)

Table S1 **Patient characteristics.** *”Patients 1” are all CD34+ patients. “Patients 2” are all patients were accurate discrimination between HSC and pLSC was enabled using our extensive gating strategy. “Patients 3” are CR patients with MRD and pLSC data to enlarge the total patient group as shown in Figure 7.
(DOCX)

Table S2 **FSC and SSC position relative to lymphocytes.** FSC, forward scatter; SSC, side scatter; HSC hematopoietic stem cells; pLSC, putative leukemia stem cell; NA, not applicable. * FSC and SSC values relative to those of lymphocytes present in the same sample.
(DOCX)

Table S3 **Gating details of 117 patients with a secondary gating strategy to define pLSC and HSC at diagnosis AML.**
(DOCX)

Table S4 **Number of patients for different strategies in 250 CD34+ AML cases.** *>20% aberrant marker expression was considered substantial to identify directly at least a substantial part of the pLSC population (179/250 patients; rows 3 and 4). In 102/179 patients (41% of all 250 CD34+ patients, row 3), pLSC frequencies may be under-estimated since additional gating strategy (with FSC/SSC etc, referred to in columns 3–7) was not possible, probably leaving part of marker negative pLSCs unidentified. In 77 of these 179 patients, an additional gating step could be performed (FSC/SSC etc, see row 4), allowing a more accurate assessment of both pLSC and HSC frequencies. #: <20% aberrant marker expression (71/250 cases) is shown in rows 5 and 6. In 31 cases (12%) only inadequate LSC assessment was possible (row 5). However, in 40 of these 71 cases HSCs could still be distinguished from pLSCs with the use of secondary parameters (row 6). Highly adequate LSC assessment, using both aberrant marker expression and secondary parameters was thus possible in 77+40 cases (47%). Columns show parameters/plots used to distinguish HSCs from pLSCs.
(DOCX)

Table S5 **Multi-lineage engraftment of marker negative FSC/SSC^low^ (CD34^high^) CD34+CD38- cells present in AML.** * in the missing mouse, engraftment could not be assessed since this mouse died before examination was possible. # In the missing mouse, no human engraftment was detected. In terms of leukemic engraftment our results also confirmed the observation of Bonnet's group that purified CD34+CD38+ and CD34- were able to engraft be it in our case after injection of high cell numbers. CD34+CD38-/CLL-1+ in pts 1 and 2 (40,000 and 130,000 cells, respectively) CD34+CD38-/CLL-1-/FSC^ high^ CD34^low^ in pt 1 (6,000 cells) CD34+CD38+ in pts 2, 4, 5, 6 (high cell numbers, 100,000-10^6^ injected in pts 2, 4, 6 and 1,000 in pt 5) CD34- in pts 2 and 5 (high cell numbers injected:100,000-10^6^).
(DOCX)

Table S6 **Cut-off values in the CD34+CD38-, CD34+CD38+ and CD34- cell compartment at diagnosis to identify patient groups with different survival.** *p-values refer to significance of differences in RFS between patients above and patients below the indicated cut-offs.
(DOCX)

Table S7 **Relative risk of relapse determined for pLSC- and pLSC+ patients at follow up defined by different cut-off points.** Not shown in the Table: for RFS and OS, without use of cut-offs, Cox regression analysis showed a strong significant inverse correlation between pLSC percentage and **RFS** after 1^st^ cycle (n = 71, RR = 2.4, 95%CI:1.3–4.6, p = 0.008), 2^nd^ cycle (n = 77, RR = 2.5, 95%CI:1.7–3.7, p<0.001) and consolidation cycle (n = 48, RR = 3.0, 95%CI:1.4–6.2, p = 0.004). For **OS**, these figures were RR = 1.8 (p = 0.04), RR = 2.7 (p<0.001) and RR = 2.0 (p = 0.07). Hereafter different cut offs were applied for risk on relapse. RR, relative risk of relapse using these different cut offs. Cut-offs of 0.0003% (3 in a million, 1^st^ cycle) and 0.0001% (2nd and consolidation cycle) were used for relapse-free survival **(RFS)** in Kaplan-Meier analyses shown in Figure 6. With these cut-offs median overall survival **(OS**, not shown in Figure 6 **)** was not reached (>42 months) for pLSC+ patients after 1^st^ cycle, but more patients survived in the pLSC- group (p = 0.002). After 2^nd^ cycle median **OS** in pLSC- group was>45 months versus 35 months in pLSC+ group (p<0.001). After consolidation cycle these figures were both>37 months (p = 0.05).
(DOCX)

Table S8 **Multivariate analysis^#^ for impact of pLSC frequency on RFS.** ^#^ in univariate analyses performed on 162 CD34+ patients, cytogenetic/molecular risk (p = 0.001, n = 162), number of chemotherapy cycles needed to achieve CR (p<0.001, n = 162), and WBC count at diagnosis (p = 0.002, n = 162) were significant. Other factors showed a trend: NPM1 mutation (p = 0.093, n = 140) and EVI-1 (p = 0.18, n = 140). n.r. not relevant since all evaluated patients achieved CR after first cycle RR, relative risk of relapse. * only the most optimal cut-offs (0.0003% after 1^st^ cycle and 0.0001% after 2^nd^ and consolidation cycle) are shown.
(DOCX)

Text S1 **Supporting text.**
(DOCX)
