# Supplementary figures and images for: Leukemic Stem Cell Frequency: A Strong Biomarker for Clinical Outcome in Acute Myeloid Leukemia
Source: PLoS One. 2014 Sep 22;9(9):e107587. doi: 10.1371/journal.pone.0107587 (PMC4171508; doi:10.1371/journal.pone.0107587)

## Slide 1
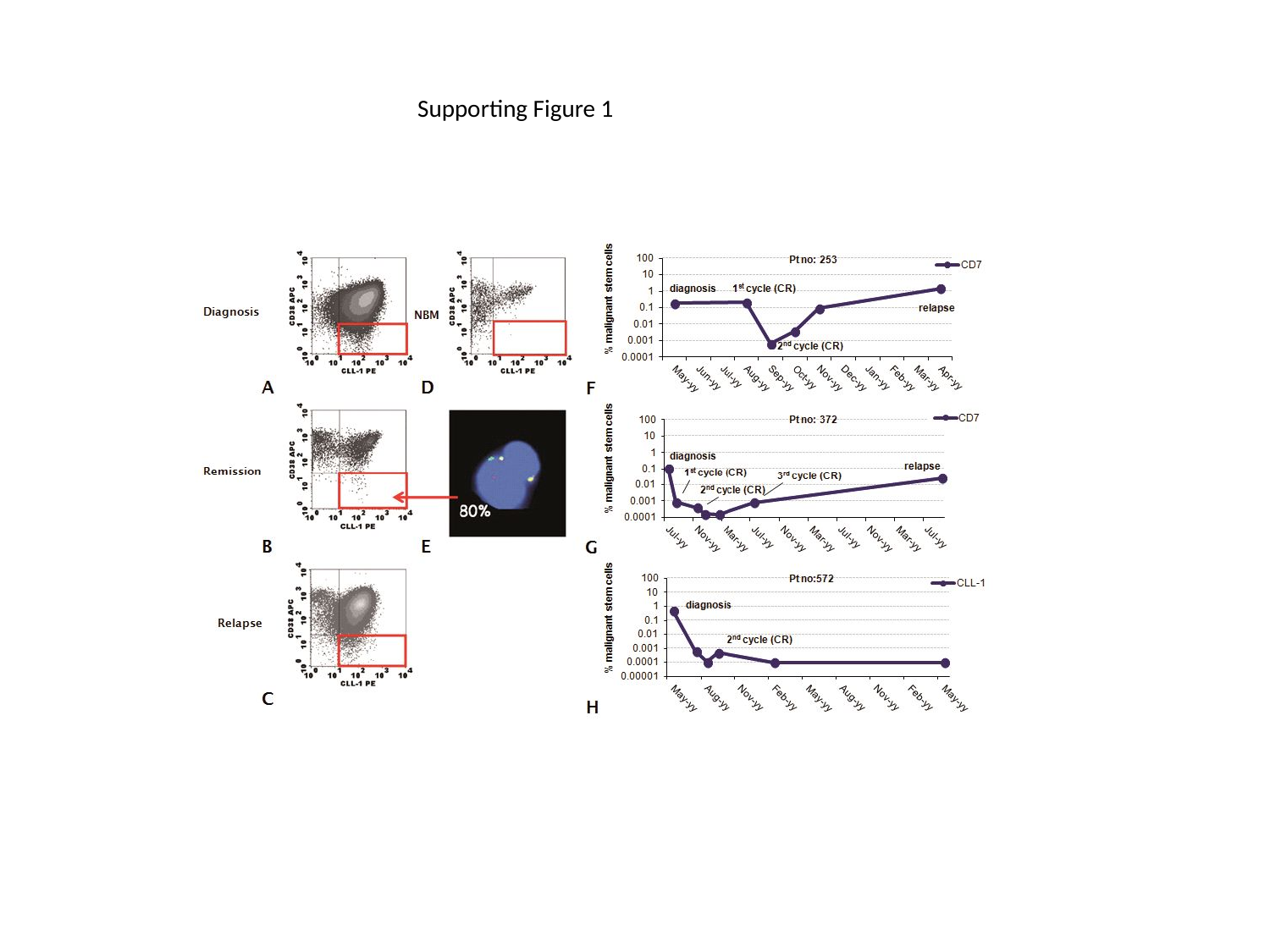

Supporting Figure 1

Supplement: Figure S1 — pLSCs monitoring during sequential BM sampling. A patient (243) with AML positive for a t(8;21) showed CLL-1 expression in the CD34+CD38- compartment (A), while normal BM misses CLL-1+ CD34+CD38- cells [1]. In CR, CLL-1+ CD34+CD38- cells (B) were sorted and assessed for t(8;21): mainly neoplastic cells were present (E). Normal CD34+CD38-CLL-1 negative cells were almost completely absent here. Shown in F-H are examples of sequential monitoring in three cases with increasing periods of complete remission until relapse (F, pt 253; G, pt 372) and in continuous remission (H, pt 572). Note the increase of pLSC frequency preceding relapse (F, G). (PPTX) [file pone.0107587.s001.pptx]
