# Supplementary material for: Leukemic Stem Cell Frequency: A Strong Biomarker for Clinical Outcome in Acute Myeloid Leukemia
Source: PLoS One. 2014 Sep 22;9(9):e107587. doi: 10.1371/journal.pone.0107587 (PMC4171508; doi:10.1371/journal.pone.0107587)
Supplement: Table S1 — Patient characteristics. *”Patients 1” are all CD34+ patients. “Patients 2” are all patients were accurate discrimination between HSC and pLSC was enabled using our extensive gating strategy. “Patients 3” are CR patients with MRD and pLSC data to enlarge the total patient group as shown in Figure 7. (DOCX) [file pone.0107587.s002.docx]

| **Table S1. Patient characteristics** | | | | | | |
| --- | --- | --- | --- | --- | --- | --- |
|  | **Patients 1*** | **%** | **Patients 2*** | **%** | **Patients 3*** | **%** |
| **Total** | 250 |  | 117 |  | 23 |  |
| **Male/Female** | 133/117 | 53/47 | 64/53 | 54/45 | 13/10 | 57/43 |
| **Age** |  |  |  |  |  |  |
| **HOVON/SAKK treatment study** | 42a |  | 42a |  | 92 |  |
| **median (range)** | 50 (18-60) |  | 49 (18-60) |  | 53 (18-64) |  |
| **WBC at diagnosis ×10^9^/l** |  |  |  |  |  |  |
| **≤20** | 158 | 63 | 72 | 61 | 13 | 57 |
| **20-100** | 58 | 23 | 31 | 26 | 7 | 30 |
| **>100** | 34 | 14 | 14 | 12 | 3 | 13 |
| **FAB** |  |  |  |  |  |  |
| **M0** | 29 | 12 | 8 | 7 | 2 | 9 |
| **M1** | 54 | 22 | 34 | 29 | 4 | 17 |
| **M2** | 76 | 30 | 39 | 33 | 7 | 30 |
| **M4** | 33 | 13 | 12 | 10 | 2 | 9 |
| **M5** | 16 | 6 | 7 | 6 | 4 | 17 |
| **M6** | 8 | 3 | 5 | 4 | 1 | 4 |
| **M7** | 1 | 0.4 | - | - |  |  |
| **RAEB** | 10 | 4 | 1 | 1 | 1 | 4 |
| **RAEB-t** | 14 | 6 | 5 | 4 |  |  |
| **Not classified** | 9 | 3 | 6 | 5 | 2 | 9 |
| **Molecular/cytogenetic risk group(%)** |  |  |  |  |  |  |
| **Favourable** | 46 | 19 | 27 | 23 | 1 | 4 |
| **Intermediate** | 34 | 13 | 16 | 14 | 11 | 48 |
| **Poor** | 105 | 42 | 52 | 44 | 8 | 35 |
| **Very poor** | 65 | 26 | 22 | 19 | 3 | 13 |
| **CR reached** |  |  |  |  |  |  |
| **Never CR** | 66 | 26 | 23 | 20 | 0 | 0 |
| **After cycle 1** | 124 | 50 | 67 | 57 | 19 | 83 |
| **After cycle 2** | 47 | 19 | 22 | 19 | 4 | 17 |
| **Later** | 13 | 5 | 5 | 4 | - | - |
| **FLT-3** |  |  |  |  |  |  |
| **wt** | 164 | 66 | 77 | 66 | 14 | 61 |
| **ITD** | 33 | 13 | 20 | 17 | 4 | 17 |
| **Missing** | 53 | 21 | 20 | 17 | 5 | 22 |
