# Supplementary material for: Leukemic Stem Cell Frequency: A Strong Biomarker for Clinical Outcome in Acute Myeloid Leukemia
Source: PLoS One. 2014 Sep 22;9(9):e107587. doi: 10.1371/journal.pone.0107587 (PMC4171508; doi:10.1371/journal.pone.0107587)
Supplement: Table S2 — FSC and SSC position relative to lymphocytes. FSC, forward scatter; SSC, side scatter; HSC hematopoietic stem cells; pLSC, putative leukemia stem cell; NA, not applicable. * FSC and SSC values relative to those of lymphocytes present in the same sample. (DOCX) [file pone.0107587.s003.docx]

| **Table S2. FSC and SSC position relative to lymphocytes** | | | | |
| --- | --- | --- | --- | --- |
| **BM source** | **Ratios FSC and SSC***  **[median (range) number]** | | | |
|  | ***HSC*** | | ***pLSC*** | |
|  | FSC | SSC | FSC | SSC |
| **CD34+ AML**  **n=10** | 1.19 (1.08-1.34) | 1.30 (1.03-1.65) | 1.69 (1.56-1.79) | 1.93 (1.73-2.94) |
| **CD34- AML**  **n=25** | 1.21 (1.04-1.36) | 1.17 (0.91-1.51) | No CD34+CD38- pLSC present | No CD34+CD38- pLSC present |
| **Normal BM**  **n=8** | 1.42 (1.32-1.56) | 1.40 (1.25 – 1.56) | NA | NA |
|  | | | | |
