# Supplementary material for: Leukemic Stem Cell Frequency: A Strong Biomarker for Clinical Outcome in Acute Myeloid Leukemia
Source: PLoS One. 2014 Sep 22;9(9):e107587. doi: 10.1371/journal.pone.0107587 (PMC4171508; doi:10.1371/journal.pone.0107587)
Supplement: Table S3 — Gating details of 117 patients with a secondary gating strategy to define pLSC and HSC at diagnosis AML. (DOCX) [file pone.0107587.s004.docx]

| **Table S3. Gating details of 117 patients with a secondary gating strategy to define pLSC and HSC at diagnosis AML** | | | | | | | | |
| --- | --- | --- | --- | --- | --- | --- | --- | --- |
| **Study nr** | **%CD34** | **Nr of aberrancies** | **Aberrancy for primary gating** | **% expression** | **Primary gating** | **Secondary gating** | **%HSC** | **%pLSC** |
| 2005 | 19.05 | 0 | CLL-1 | 17.21 | CLL-1/FSC | FSC | 1.98 | 98.02 |
| 2009 | 44.76 | 0 | CLL-1 | 16.82 | CLL-1/FSC | FSC | 0.00 | 100.00 |
| 2018 | 37.32 | 2 | CD56 | 82.46 | CD56 | CD34 | 8.16 | 91.84 |
| 2019 | 31.19 | 2 | CD36 | 94.44 | CD36 | FSC/SSC | 5.56 | 94.44 |
| 2025 | 44.11 | 1 | CLL-1 | 62.22 | CLL-1 | CD34/FSC | 41.18 | 58.82 |
| 2027 | 25.98 | 1 | CD22 | 31.58 | CD22 | CD34 | 47.37 | 52.63 |
| 2045 | 21.60 | 3 | CLL-1 | 54.55 | CLL-1 | FSC/CD34 | 26.32 | 73.68 |
| 2046 | 1.06 | 0 | CLL-1 | 6.12 | CLL-1/FSC/CD34 | CD34/FSC | 86.11 | 13.89 |
| 2047 | 20.61 | 1 | CLL-1 | 59.58 | CLL-1 | FSC | 3.29 | 96.71 |
| 2048 | 4.05 | 4 | CD56 | 77.18 | CD56 | FSC/SSC | 2.87 | 85.21 |
| 2049 | 1.49 | 2 | CD7 | 68.57 | CD7 | FSC/SSC | 21.43 | 78.57 |
| 2050 | 18.32 | 2 | CLL-1 | 43.45 | CLL-1 | FSC | 33.70 | 66.30 |
| 2052 | 28.07 | 0 | CD7 | 19.70 | CD7/FSC | FSC/CD34 | 41.94 | 58.06 |
| 2057 | 69.21 | 1 | CLL-1 | 94.78 | CLL-1 | FSC | 5.22 | 94.78 |
| 2059 | 3.19 | 1 | CLL-1 | 80.85 | CLL-1 | FSC/CD34 | 0.00 | 100.00 |
| 2061 | 65.16 | 0 | CLL-1 | 17.39 | CLL-1/FSC | FSC | 100.00 | 0.00 |
| 2062 | 29.70 | 0 | CLL-1 | 0.00 | FSC | FSC | 100.00 | 0.00 |
| 2066 | 52.09 | 1 | CD11b | 87.91 | CD11b | FSC | 0.00 | 100.00 |
| 2071 | 18.51 | 1 | CLL-1 | 61.11 | CLL-1 | FSC/CD34 | 38.89 | 61.11 |
| 2077 | 24.32 | 2 | CD22 | 41.61 | CD22 | CD34 | 71.43 | 28.57 |
| 2080 | 49.74 | 2 | CLL-1 | 74.32 | CLL-1 | FSC | 0.83 | 99.17 |
| 2087 | 81.10 | 1 | CD7 | 88.71 | CD7 | CD34 | 10.19 | 89.81 |
| 2093 | 51.40 | 0 | CLL-1 | 2.60 | CLL-1/FSC/CD34 | CD34 | 43.42 | 56.58 |
| 2096 | 65.75 | 1 | CD19 | 69.59 | CD19 | FSC/SSC/CD45/CD34 | 18.25 | 81.75 |
| 2098 | 69.25 | 0 | CLL-1 | 2.46 | CLL-1/FSC | FSC/CD34/CD45 | 77.29 | 22.71 |
| 2102 | 25.68 | 1 | CLL-1 | 68.57 | CLL-1 | CD34 | 17.96 | 82.04 |
| 2120 | 12.02 | 0 | CLL-1 | 6.59 | CLL-1/FSC/CD34 | FSC | 40.25 | 59.75 |
| 2122 | 48.36 | 3 | CD22 | 40.40 | CD22 | CD34/FSC | 40.40 | 59.60 |
| 2124 | 11.12 | 1 | CLL-1 | 37.50 | CLL-1 | FSC/CD45 | 62.50 | 37.50 |
| 2137 | 44.63 | 1 | CD7 | 25.00 | CD7 | FSC/CD45 | 33.33 | 66.67 |
| 2145 | 50.02 | 1 | CLL-1 | 74.68 | CLL-1 | FSC | 11.11 | 88.89 |
| 2156 | 1.04 | 0 | CLL-1 | 17.17 | CLL-1/FSC | FSC/CD34 | 1.43 | 98.57 |
| 2157 | 20.27 | 2 | CLL-1 | 67.88 | CLL-1 | FSC/SSC | 30.90 | 69.10 |
| 2158 | 4.63 | 0 | CLL-1 | 7.60 | CLL-1/CD34 | CD34/FSC | 4.32 | 95.68 |
| 2161 | 5.94 | 2 | CLL-1 | 81.60 | CLL-1 | FSC | 3.88 | 96.12 |
| 2166 | 4.02 | 1 | CLL-1 | 20.75 | CLL-1 | FSC | 51.25 | 48.75 |
| 2167 | 27.89 | 0 | CLL-1 | 0.00 | FSC/CD34 | CD34 | 77.78 | 22.22 |
| 2168 | 45.31 | 2 | CD7 | 50.85 | CD7 | FSC/SSC | 68.00 | 32.00 |
| 2171 | 28.57 | 0 | CLL-1 | 1.11 | CLL-1/FSC | FSC | 98.77 | 1.23 |
| 2175 | 34.34 | 2 | CLL-1 | 77.59 | CLL-1 | FSC | 22.41 | 77.59 |
| 2178 | 6.54 | 0 | CLL-1 | 3.60 | CLL-1/FSC | FSC | 63.91 | 36.09 |
| 2182 | 23.21 | 0 | CLL-1 | 0.00 | FSC/CD34 | FSC/CD34/CD45 | 85.29 | 14.71 |
| 2186 | 9.13 | 1 | CD7 | 52.48 | CD7 | FSC/SSC | 3.42 | 96.58 |
| 2187 | 6.26 | 3 | CLL-1 | 36.14 | CLL-1 | FSC/SSC | 62.65 | 37.35 |
| 2200 | 26.73 | 0 | CLL-1 | 17.48 | CLL-1/FSC | FSC | 3.07 | 96.93 |
| 2205 | 18.34 | 1 | CLL-1 | 66.67 | CLL-1 | FSC/SSC | 33.30 | 66.70 |
| 2212 | 78.76 | 2 | CD19 | 95.04 | CD19 | FSC/SSC | 0.71 | 99.29 |
| 2216 | 1.11 | 1 | CD11b | 50.56 | CD11b | CD34 | 4.12 | 95.88 |
| 2217 | 3.69 | 2 | CD11b | 70.20 | CD11b | FSC/SSC | 13.91 | 86.09 |
| 2224 | 14.04 | 2 | CD7 | 31.58 | CD7 | FSC/SSC | 31.58 | 68.42 |
| 2230 | 17.53 | 3 | CD56 | 90.92 | CD56 | FSC | 3.74 | 96.26 |
| 2243 | 34.88 | 1 | CLL-1 | 29.41 | CLL-1 | FSC | 3.41 | 96.59 |
| 2247 | 67.83 | 1 | CD7 | 44.62 | CD7 | CD34/FSC/SSC | 4.27 | 95.73 |
| 2248 | 24.62 | 1 | CLL-1 | 82.88 | CLL-1 | FSC/CD45 | 3.60 | 96.40 |
| 2251 | 2.69 | 1 | CD7 | 86.68 | CD7 | FSC/CD34 | 0.90 | 99.10 |
| 2254 | 39.39 | 0 | CLL-1 | 8.44 | FSC | FSC | 11.06 | 88.94 |
| 2258 | 55.05 | 2 | CLL-1 | 88.84 | CLL-1 | CD34 | 5.90 | 94.10 |
| 2267 | 83.11 | 1 | CLL-1 | 45.31 | CLL-1 | FSC/CD45 | 5.70 | 94.30 |
| 2270 | 23.58 | 0 | CLL-1 | 5.71 | CLL-1/CD45 | FSC/CD45 | 34.29 | 65.71 |
| 2281 | 22.95 | 3 | CD56 | 38.46 | CD56 | CD34 | 23.08 | 76.92 |
| 2283 | 65.77 | 1 | CD7 | 20.43 | CD7 | FSC | 0.00 | 100.00 |
| 2285 | 11.30 | 4 | CD56 | 99.16 | CD56 | CD34/FSC/SSC | 0.43 | 99.99 |
| 2292 | 4.65 | 2 | CLL-1 | 64.83 | CLL-1 | CD34/FSC | 1.32 | 98.68 |
| 2298 | 58.03 | 1 | CLL-1 | 22.50 | CLL-1 | FSC | 87.50 | 12.50 |
| 2309 | 71.24 | 0 | CLL-1 | 0.00 | FSC/CD34 | FSC/CD34 | 91.49 | 8.51 |
| 2310 | 1.85 | 1 | CLL-1 | 34.21 | CLL-1 | CD34/CD45 | 57.89 | 42.11 |
| 2321 | 10.50 | 1 | CLL-1 | 27.33 | CLL-1 | FSC/CD34 | 12.50 | 87.50 |
| 2326 | 4.62 | 0 | CLL-1 | 9.43 | CLL-1/FSC | FSC/CD34 | 8.86 | 91.14 |
| 2328 | 26.96 | 0 | CLL-1 | 18.18 | CLL-1/CD34 | CD34 | 77.78 | 22.22 |
| 2334 | 13.13 | 2 | CD7 | 22.10 | CD7 | FSC | 19.89 | 80.11 |
| 2336 | 57.06 | 0 | CLL-1 | 9.87 | CLL-1/CD45 | FSC/CD45 | 5.89 | 94.11 |
| 2349 | 45.40 | 0 | CD7 | 3.23 | CD7/FSC | FSC | 100.00 | 0.00 |
| 2356 | 29.93 | 1 | CLL-1 | 76.27 | CLL-1 | CD34 | 6.78 | 93.22 |
| 2359 | 39.19 | 2 | CD19 | 33.96 | CD19 | FSC/CD45 | 43.40 | 56.60 |
| 2362 | 76.04 | 2 | CLL-1 | 57.14 | CLL-1 | FSC/SSC | 16.67 | 83.33 |
| 2363 | 10.74 | 0 | CLL-1 | 9.88 | CLL-1/FSC | FSC | 60.00 | 40.00 |
| 2364 | 68.45 | 0 | CLL-1 | 2.12 | CLL-1/CD34/CD45 | CD34/CD45 | 7.20 | 92.80 |
| 2365 | 31.19 | 0 | CD7 | 6.25 | CD7/FSC | FSC/CD34 | 62.50 | 37.50 |
| 2368 | 61.88 | 0 | CD56 | 6.19 | CD56/FSC | FSC | 2.15 | 97.85 |
| 2369 | 70.96 | 1 | CD7 | 27.21 | CD7 | FSC | 72.79 | 27.21 |
| 2373 | 19.62 | 0 | CLL-1 | 16.90 | CLL-1/FSC | FSC/CD45 | 65.52 | 34.48 |
| 2377 | 18.72 | 0 | CLL-1 | 6.79 | CLL-1/FSC/CD34 | FSC/CD34 | 96.03 | 3.97 |
| 2383 | 56.07 | 2 | CD7 | 56.05 | CD7 | FSC | 13.84 | 86.16 |
| 2393 | 24.80 | 2 | CLL-1 | 31.15 | CLL-1 | FSC | 10.71 | 89.29 |
| 2397 | 14.15 | 1 | CLL-1 | 75.44 | CLL-1 | CD34 | 3.31 | 96.69 |
| 2398 | 72.79 | 1 | CLL-1 | 98.35 | CLL-1 | FSC/CD34/CD45 | 1.89 | 98.11 |
| 2414 | 49.61 | 0 | CD7 | 4.86 | CD7/FSC/CD34 | FSC/CD34 | 84.62 | 15.38 |
| 2417 | 1.48 | 1 | DR- | 72.97 | DR- ZW | CD34 | 80.00 | 20.00 |
| 2419 | 73.77 | 2 | CD11b | 95.83 | CD11b | CD34 | 5.64 | 94.36 |
| 2423 | 7.85 | 0 | CLL-1 | 7.69 | CLL-1/FSC | FSC/CD34 | 71.43 | 28.57 |
| 2424 | 66.19 | 1 | CLL-1 | 29.79 | CLL-1 | FSC/CD45 | 56.34 | 43.66 |
| 2425 | 13.21 | 1 | CLL-1 | 59.15 | CLL-1 | CD34 | 8.45 | 91.55 |
| 2433 | 12.24 | 2 | CD56 | 83.66 | CD56 | FSC/CD34 | 1.73 | 98.27 |
| 2439 | 18.77 | 1 | CLL-1 | 37.64 | CLL-1 | FSC/CD34 | 37.93 | 62.07 |
| 2446 | 56.32 | 0 | CD7 | 0.00 | FSC | FSC | 100.00 | 0.00 |
| 2447 | 19.29 | 2 | CD7 | 26.09 | CD7 | FSC | 38.10 | 61.90 |
| 2449 | 20.53 | 0 | CLL-1 | 3.28 | CLL-1/CD34 | CD34 | 5.02 | 94.98 |
| 2450 | 2.26 | 1 | CLL-1 | 32.58 | CLL-1 | CD34 | 11.64 | 88.36 |
| 2452 | 8.37 | 2 | CD2 | 88.84 | CD2 | CD34 | 4.56 | 95.44 |
| 2453 | 5.16 | 0 | CLL-1 | 7.14 | CLL-1/CD34 | CD34 | 23.81 | 76.19 |
| 2459 | 2.28 | 1 | CLL-1 | 86.55 | CLL-1 | CD34 | 7.58 | 92.42 |
| 2461 | 33.73 | 0 | CLL-1 | 0.00 | FSC/CD34 | CD34 | 40.00 | 60.00 |
| 2464 | 1.42 | 0 | CLL-1 | 2.29 | CLL-1/FSC | FSC | 55.28 | 44.72 |
| 2466 | 34.24 | 2 | CD36 | 78.18 | CD36 | FSC/SSC/CD45 | 2.28 | 97.72 |
| 2467 | 58.13 | 1 | CLL-1 | 33.33 | CLL-1 | FSC/CD45 | 61.11 | 38.89 |
| 2468 | 12.14 | 0 | CLL-1 | 1.31 | CD34 | CD34 | 16.07 | 83.93 |
| 2469 | 2.05 | 1 | CLL-1 | 39.60 | CLL-1 | CD34 | 10.56 | 89.44 |
| 2473 | 21.67 | 0 | CLL-1 | 7.69 | CLL-1/FSC/CD34 | FSC/CD34 | 100.00 | 0.00 |
| 2474 | 30.71 | 4 | CD7 | 74.84 | CD7 | CD34 | 3.42 | 93.74 |
| 2475 | 8.50 | 1 | CD7 | 56.38 | CD7 | CD34/FSC | 15.96 | 84.04 |
| 2477 | 32.31 | 1 | CLL-1 | 42.94 | CLL-1 | CD34/CD45 | 13.95 | 86.05 |
| 2487 | 5.98 | 0 | CLL-1 | 0.00 | FSC | FSC | 83.33 | 16.67 |
| 2490 | 11.41 | 3 | CD22 | 99.29 | CD22 | CD34 | 0.20 | 99.80 |
| 2493 | 11.77 | 3 | CD11b | 94.96 | CD11b | CD34 | 1.94 | 98.06 |
| 2494 | 1.28 | 0 | CLL-1 | 6.70 | CLL-1/FSC | FSC | 35.38 | 64.62 |
| 2507 | 42.71 | 3 | CLL-1 | 52.54 | CLL-1 | CD34 | 25.42 | 74.58 |
| 2509 | 19.55 | 1 | CLL-1 | 54.91 | CLL-1 | CD45/FSC | 3.35 | 96.65 |
|  |  |  |  |  |  |  |  |  |
| **Median** |  |  |  |  |  |  | **18** | **82** |
| (range) |  |  |  |  |  |  | (0-100) | (0-100) |
