# Supplementary material for: Leukemic Stem Cell Frequency: A Strong Biomarker for Clinical Outcome in Acute Myeloid Leukemia
Source: PLoS One. 2014 Sep 22;9(9):e107587. doi: 10.1371/journal.pone.0107587 (PMC4171508; doi:10.1371/journal.pone.0107587)
Supplement: Table S4 — Number of patients for different strategies in 250 CD34+ AML cases. *>20% aberrant marker expression was considered substantial to identify directly at least a substantial part of the pLSC population (179/250 patients; rows 3 and 4). In 102/179 patients (41% of all 250 CD34+ patients, row 3), pLSC frequencies may be under-estimated since additional gating strategy (with FSC/SSC etc, referred to in columns 3–7) was not possible, probably leaving part of marker negative pLSCs unidentified. In 77 of these 179 patients, an additional gating step could be performed (FSC/SSC etc, see row 4), allowing a more accurate assessment of both pLSC and HSC frequencies. #: <20% aberrant marker expression (71/250 cases) is shown in rows 5 and 6. In 31 cases (12%) only inadequate LSC assessment was possible (row 5). However, in 40 of these 71 cases HSCs could still be distinguished from pLSCs with the use of secondary parameters (row 6). Highly adequate LSC assessment, using both aberrant marker expression and secondary parameters was thus possible in 77+40 cases (47%). Columns show parameters/plots used to distinguish HSCs from pLSCs. (DOCX) [file pone.0107587.s005.docx]

| **Table S4. Number of patients for different strategies in 250 CD34+ AML cases** | | | | | | |
| --- | --- | --- | --- | --- | --- | --- |
| **AML type** | **N_tot_** | **Number of patients with secondary gating options possible** | | | | |
|  |  | **FSC/SSC** | **FSC/SSC and CD34/SSC** | **CD34/SSC** | **CD34/SSC and CD45/SSC** | **FSC/SSC and CD45/SSC** |
| **CD34+CD38-with aberrant marker expression >20%* n=179** | 102 | - | - | - | - | - |
|  | 77 | 30 | 16 | 21 | 2 | 8 |
| **CD34+CD38-with aberrant marker expression <20%^#^**  **n=71** | 31 | - | - | - | - | - |
|  | 40 | 16 | 12 | 7 | 1 | 4 |
