# Supplementary material for: Leukemic Stem Cell Frequency: A Strong Biomarker for Clinical Outcome in Acute Myeloid Leukemia
Source: PLoS One. 2014 Sep 22;9(9):e107587. doi: 10.1371/journal.pone.0107587 (PMC4171508; doi:10.1371/journal.pone.0107587)
Supplement: Table S5 — Multi-lineage engraftment of marker negative FSC/SSClow (CD34high) CD34+CD38- cells present in AML. * in the missing mouse, engraftment could not be assessed since this mouse died before examination was possible. # In the missing mouse, no human engraftment was detected. In terms of leukemic engraftment our results also confirmed the observation of Bonnet's group that purified CD34+CD38+ and CD34- were able to engraft be it in our case after injection of high cell numbers. CD34+CD38-/CLL-1+ in pts 1 and 2 (40,000 and 130,000 cells, respectively) CD34+CD38-/CLL-1-/FSC high CD34low in pt 1 (6,000 cells) CD34+CD38+ in pts 2, 4, 5, 6 (high cell numbers, 100,000-106 injected in pts 2, 4, 6 and 1,000 in pt 5) CD34- in pts 2 and 5 (high cell numbers injected:100,000-106). (DOCX) [file pone.0107587.s006.docx]

| **Table S5. Multi-lineage engraftment of marker negative FSC/SSC^low^ (CD34^high^) CD34+CD38- cells present in AML** | | | | |
| --- | --- | --- | --- | --- |
| **Patient** | **Primary gating** | **Secondary gating** | **# cells** | **Engraftment type (%)** |
| **598** | CD34+CD38-/ CLL-1- | FSC/SSC^low^ and CD34^high^ | 4400 | Multilineage 1/2* (0.3%) |
| **1034** | CD34+CD38-/ CLL-1- | FSC/SSC^low^ and CD34^high^ | 3800 | Multilineage 2/2 (4%;6%) |
| **661** | CD34+CD38-/ CD11b- | FSC/SSC^low^ | 12000 | Multilineage 2/2 (6%;1.4%) |
| **423** | CD34+CD38-/ CLL-1- | FSC/SSC^low^ and CD34^high^ | 1400 | Multilineage 1/2* (2.8%) |
| **928** | CD34+CD38-/ CD13+ | FSC/SSC^low^ and CD34^high^ | 5500 | Multilineage 1/2^#^ (0.1%) |
| **641** | CD34+CD38-/ CD22- | FSC/SSC^low^ | 20000 | None 1/2* |
