# Supplementary material for: Leukemic Stem Cell Frequency: A Strong Biomarker for Clinical Outcome in Acute Myeloid Leukemia
Source: PLoS One. 2014 Sep 22;9(9):e107587. doi: 10.1371/journal.pone.0107587 (PMC4171508; doi:10.1371/journal.pone.0107587)
Supplement: Table S6 — Cut-off values in the CD34+CD38-, CD34+CD38+ and CD34- cell compartment at diagnosis to identify patient groups with different survival. *p-values refer to significance of differences in RFS between patients above and patients below the indicated cut-offs. (DOCX) [file pone.0107587.s007.docx]

| **Table S6: Cut-off values in the CD34+CD38-, CD34+CD38+ and CD34- cell compartment at diagnosis to identify patient groups with different survival** | | | | | | | |
| --- | --- | --- | --- | --- | --- | --- | --- |
| **A. CD34+CD38-** |  |  |  |  |  |  |  |
| **cut-off** | **0.002%** | **0.005%** | **0.01%** | **0.03%** | **0.1%** | **0.2%** | **1%** |
| **Patients (n) above cutoff** | 60 | 51 | 40 | 36 | 25 | 20 | 7 |
| **Patients (n) below cutoff** | 10 | 19 | 30 | 34 | 45 | 50 | 63 |
| **p-value*** | 0.072 | 0.002 | 0.030 | 0.015 | 0.001 | 0.006 | 0.617 |
| **B. CD34+CD38+** |  |  |  |  |  |  |  |
| **cut-off** | **2%** | **5%** | **15%** | **25%** | **30%** | **40%** | **60%** |
| **Patients (n) above cutoff** | 64 | 58 | 41 | 33 | 29 | 17 | 8 |
| **Patients (n) below cutoff** | 6 | 12 | 29 | 37 | 41 | 53 | 64 |
| **p-value*** | 0.411 | 0.204 | 0.750 | 0.987 | 0.941 | 0.108 | 0.314 |
| **C. CD34-** |  |  |  |  |  |  |  |
| **cut-off** | **0.1%** | **0.5%** | **1%** | **3.5%** | **5%** | **15%** | **30%** |
| **Patients (n) above cutoff** | 64 | 52 | 45 | 32 | 28 | 13 | 5 |
| **Patients (n) below cutoff** | 6 | 18 | 25 | 38 | 42 | 57 | 65 |
| **p-value*** | 0.655 | 0.940 | 0.237 | 0.835 | 0.613 | 0.992 | 0.446 |
