# Supplementary material for: Leukemic Stem Cell Frequency: A Strong Biomarker for Clinical Outcome in Acute Myeloid Leukemia
Source: PLoS One. 2014 Sep 22;9(9):e107587. doi: 10.1371/journal.pone.0107587 (PMC4171508; doi:10.1371/journal.pone.0107587)
Supplement: Table S7 — Relative risk of relapse determined for pLSC- and pLSC+ patients at follow up defined by different cut-off points. Not shown in the Table: for RFS and OS, without use of cut-offs, Cox regression analysis showed a strong significant inverse correlation between pLSC percentage and RFS after 1st cycle (n = 71, RR = 2.4, 95%CI:1.3–4.6, p = 0.008), 2nd cycle (n = 77, RR = 2.5, 95%CI:1.7–3.7, p<0.001) and consolidation cycle (n = 48, RR = 3.0, 95%CI:1.4–6.2, p = 0.004). For OS, these figures were RR = 1.8 (p = 0.04), RR = 2.7 (p<0.001) and RR = 2.0 (p = 0.07). Hereafter different cut offs were applied for risk on relapse. RR, relative risk of relapse using these different cut offs. Cut-offs of 0.0003% (3 in a million, 1st cycle) and 0.0001% (2nd and consolidation cycle) were used for relapse-free survival (RFS) in Kaplan-Meier analyses shown in Figure 6. With these cut-offs median overall survival (OS, not shown in Figure 6 ) was not reached (>42 months) for pLSC+ patients after 1st cycle, but more patients survived in the pLSC- group (p = 0.002). After 2nd cycle median OS in pLSC- group was>45 months versus 35 months in pLSC+ group (p<0.001). After consolidation cycle these figures were both>37 months (p = 0.05). (DOCX) [file pone.0107587.s008.docx]

| **Table S7. Relative risk of relapse determined for pLSC- and pLSC+ patients at follow up defined by different cut-off points.** | | | | | | | | | | | | | | | | | | |
| --- | --- | --- | --- | --- | --- | --- | --- | --- | --- | --- | --- | --- | --- | --- | --- | --- | --- | --- |
|  | **First induction cycle** | | | | | | **Second induction cycle** | | | | | | **Consolidation therapy** | | | | | |
| **Cut-off (%)** | 0.0001 | 0.0002 | 0.0003 | 0.0004 | 0.0005 | 0.001 | 0.0001 | 0.0002 | 0.0003 | 0.0004 | 0.0005 | 0.001 | 0.0001 | 0.0002 | 0.0003 | 0.0004 | 0.0005 | 0.001 |
| **pLSC- (n)** | 25 | 42 | 49 | 53 | 56 | 65 | 36 | 48 | 52 | 56 | 57 | 67 | 28 | 34 | 37 | 38 | 41 | 43 |
| **pLSC+ (n)** | 46 | 29 | 22 | 18 | 15 | 6 | 41 | 29 | 25 | 21 | 20 | 10 | 20 | 14 | 11 | 10 | 7 | 5 |
| **RR relapse** | 2.0 | 3.1 | 5 | 3.6 | 3.5 | 2.9 | 6.3 | 3.4 | 4.4 | 4.8 | 4.3 | 4 | 6.4 | 5.4 | 5.1 | 6.3 | 4.9 | 2.1 |
| **p-value** | 0.19 | 0.019 | 0.001 | 0.005 | 0.009 | 0.14 | <0.001 | <0.001 | <0.001 | <0.001 | <0.001 | 0.001 | 0.006 | 0.007 | 0.007 | 0.002 | 0.011 | 0.34 |
|  | | | | | | | | | | | | | | | | | | |
