# Supplementary material for: Leukemic Stem Cell Frequency: A Strong Biomarker for Clinical Outcome in Acute Myeloid Leukemia
Source: PLoS One. 2014 Sep 22;9(9):e107587. doi: 10.1371/journal.pone.0107587 (PMC4171508; doi:10.1371/journal.pone.0107587)
Supplement: Table S8 — Multivariate analysis# for impact of pLSC frequency on RFS. # in univariate analyses performed on 162 CD34+ patients, cytogenetic/molecular risk (p = 0.001, n = 162), number of chemotherapy cycles needed to achieve CR (p<0.001, n = 162), and WBC count at diagnosis (p = 0.002, n = 162) were significant. Other factors showed a trend: NPM1 mutation (p = 0.093, n = 140) and EVI-1 (p = 0.18, n = 140). n.r. not relevant since all evaluated patients achieved CR after first cycle RR, relative risk of relapse. * only the most optimal cut-offs (0.0003% after 1st cycle and 0.0001% after 2nd and consolidation cycle) are shown. (DOCX) [file pone.0107587.s009.docx]

| **Table S8. Multivariate analysis^#^ for impact of pLSC frequency on RFS** | | | | | | |
| --- | --- | --- | --- | --- | --- | --- |
|  | **First induction cycle** | | **Second induction cycle** | | **Consolidation therapy** | |
|  | **p-value** | **RR** | **p-value** | **RR** | **p-value** | **RR** |
| **pLSC frequency (Cut-off 0.0003%)** | **0.001** | 5.2 | * |  | * |  |
| **pLSC frequency (Cut-off 0.0001%)** | * |  | **0.001** | 5.4 | **0.008** | 6.9 |
| **Cytogenetic/Molecular risk** | 0.28 |  | 0.15 |  | 0.685 |  |
| **Cycles to CR** | n.r. |  | 0.01 | 3.0 | 0.083 |  |
| **WBC** | 0.50 |  | 0.39 |  | 0.26 |  |
