# Supplementary material for: Leukemic Stem Cell Frequency: A Strong Biomarker for Clinical Outcome in Acute Myeloid Leukemia
Source: PLoS One. 2014 Sep 22;9(9):e107587. doi: 10.1371/journal.pone.0107587 (PMC4171508; doi:10.1371/journal.pone.0107587)
Supplement: Text S1 — Supporting text. (DOCX) [file pone.0107587.s010.docx]

**Supporting text**

***Patients.*** Patient characteristics are summarized in Supplementary Table 1. Of the total group of 250 patients, 219 patients showed either aberrant marker positivity on at least 20% of the cells in the CD34+CD38- compartment (n=102), or a secondary gating step using scatter and/or CD34 and/or CD45 expression could be used to identify normal and malignant cells (n=40) or both gating strategies could be combined (n=77). For monitoring pLSCs after different courses of therapy, only patients who achieved complete remission (CR) were considered eligible (162/219). Follow-up BM was obtained in 115 cases. Detailed information regarding treatment can be found at <http://www.hovon.nl>. In brief, the majority of patients received two remission induction cycles of chemotherapy plus consolidation therapy. The first induction cycle combined Cytarabine and Idarubicine and the second induction cycle combined Cytarabine and Amsacrine. During the induction phase, patients were randomised between yes/no G-CSF (5 µg/kg). Patients with a poor prognostic karyotype at diagnosis with an available donor were transplanted in an allogeneic setting when CR was achieved after induction cycle 1. Intermediate risk patients with a matched family donor were also transplanted allogeneically, while patients with a good prognostic risk profile received a third cycle of chemotherapy (Mitoxantrone and Etoposide). All other patients, if possible, underwent stem cell mobilisation and autologous stem cell transplantation or received a third cycle of chemotherapy. CR was defined as less than 5% BM blasts detected by morphology, combined with peripheral blood recovery (PBR): ANC ≥1.0 x 10^9^/l, and platelet count ≥ 100 x 10^9^/l and no leukemic blasts in the peripheral blood.

Patients were assigned to four cytogenetic/molecular risk groups based on the following criteria: good risk patients included those positive for t(8;21) with WBC ≤ 20 x 10^9^/l, those with inv(16) or t(16;16), and those without a monosomal karyotype, but with mutated CEBPα or those with mutated NPM1/FLT3 wild type in CR after the first induction cycle. Poor risk patients were those patients not in CR after the first induction cycle and those with WBC > 100 x 10^9^/l and/or abnormal cytogenetics excluding core binding factor leukemia, monosomal karyotype, 3q26 abnormalities and EVI1. Very poor risk was defined as non-core binding factor leukemia with a monosomal karyotype or positive for EVI1 or with 3q26 abnormalities. The remaining patients were classified as Intermediate risk.

At diagnosis BM samples were used (n=186) for the flow cytometric analysis of the CD34+CD38- compartment. If this was not available then PB was used (n=62, 2 samples were of unknown origin), while at follow-up, only BM samples were used. After induction treatment (first and second cycle) and after consolidation therapy, samples were collected only in case of CR. For pLSC evaluation after consolidation therapy, samples were collected median three months after the start of consolidation therapy (range 0.5- 11.3 months).

***Flow cytometry.*** Samples were analysed using a 4-color FACSCalibur from Becton Dickinson (BD, San Jose, CA, USA) using CellQuest software. Figures 1b, 2a, and 3 were created with Infinicyt software. The core combination to measure CD34+CD38- cells was performed with the following combination of antibodies: CD34 FITC (clone 8G12, dilution 1:10, Becton Dickinson, BD), CD45 PerCp (clone 2D1, dilution 1:20, BD) and CD38 APC (clone HB7, dilution 1:50, BD). Antibodies against aberrantly expressed markers CD2 (clone MT910, dilution 1:50, DakoCytomation), CD7 (clone M-T701, dilution 1:20, BD), CD11b (clone D12, dilution 1:200, BD), CD56 (clone MY31, dilution 1:50, BD), and against CLL-1 [1], were PE labelled. Antibodies against CD15 (clone MMA, dilution 1:100, BD) and HLA-DR (L243, dilution 1:100, BD) were FITC labelled, in which cases CD34 was used in PE (clone 8G12, dilution 1:20, BD).

The gating strategy for CD34+CD38- stem cells is shown in Figure 1 (part I). Terminology used throughout the paper was FSC/SSC^high^ (meaning: both FSC and SSC were high) and FSC/SSC^low^ (both FSC and SSC low) for pLSC and HSC, respectively; both reside within a gate in literature generally referred to as SSC^dim^. Similarly, the designations CD45^high^ and CD45^low^ were used for pLSC and HSC, respectively, or HSC and LSC, respectively, depending on the characteristics of the AML case (details will be provided in the text), while both reside within a gate in literature generally referred to as CD45^dim^. FACS-sorting for molecular analysis and *in vivo* experiments was performed using cryopreserved samples on a FACSAria (BD) with FACSDiva analysis software. The procedure for sample freezing and thawing was as described before [2]. 7AAD was used in the gating strategy of frozen-thawed samples to exclude non-viable cells.

***Engraftment studies.*** Since not every AML sample was expected to engraft, samples were pre-screened for leukemia initiating capacity by injecting 5-10×10^6^ mononuclear cells intravenously [3]. Mice were evaluated for human AML engraftment after a maximum of 12 weeks, or earlier when becoming ill (hunch-back, substantial weight loss and a ruffled coat). Only the samples that initiated human leukemia engraftment, were selected for subsequent experiments. Cell fractions from these samples were sorted and injected intrafemorally. The method of intrafemoral injection was adapted from Yahata et al. [4], but in addition a 27G needle was used to make a small hole in the femur, and subsequently the cells were injected using an insulin syringe with a fixed 30G needle. Cells in PBS/0.1% HSA in a volume up to 30 µl were injected into the bone. Injections were performed under complete anaesthesia (250 µl of a ketamin 10 mg/ml / xylazin 1 mg/ml mix) and analgesia was given subcutaneously (Carprofen 4mg/kg). Mice were kept for a maximum of 12 weeks after injection of the human cells, after which they were sacrificed and analysed for engraftment. In case of illness, mice were sacrificed earlier. The screening for human cells in mouse BM was done using flowcytometry with a human PerCp labelled CD45 monoclonal antibody (clone 2D1, dilution 1:20, BD) and a murine PE-labelled antibody (clone 30-F11, dilution 1:2,000, BD-Pharmingen). Engraftment was defined as outlined under Patients, Materials and Methods in the main text.

***FISH, FLT3-ITD and NPM1 analysis.*** Mutations in *NPM1*exon 12 were analyzed via PCR on genomic DNA that was isolated from sorted cell fractions. PCR amplification was subsequently performed with the following primers: *NPM1* forward: 5′-TTAACTCTCTGGT-GGTAGAATGA-3′; *NPM1* reverse: 5′-CTGACCACCGCTACTACTATGT-3′, located in intron 11 and exon 12, respectively. Subsequent fragment analysis was performed with a tetrachlorofluorescein phosphoramidite–labeled (Biolegio, Nijmegen, The Netherlands) forward primer. Mutations detected with melting curve analysis were confirmed by bidirectional DNA sequencing on an ABI 3500 automated sequencer with the use of the BigDye terminator kit (Applied Biosystems Inc). For both FLT3 and NPM1 analysis, the bulk of AML blasts (CD34+CD38+, or with lower CD34 percentages, the CD45^dim^ fraction) was used as an internal positive control, while lymphocytes served as an internal negative control.

***Survival analysis.*** Overall survival (OS) and relapse free survival (RFS) were defined according to the landmark method: time starts at the moment of sampling and ends at time of death (OS) or moment of relapse (RFS). Event-free survival (EFS) refers to the interval from moment of sampling to the date disease progression if CR had not been achieved, the date of death, or the date of relapse. Patients without a defined event were censored in the statistical analysis at the time of last follow-up. Log-rank statistics were used to define statistical significance for the Kaplan-Meier analysis. Cox regression analysis was used to determine the relation between pLSC percentage and prognosis and was carried out on logarithmically transformed pLSC percentages to obtain a normal distribution. For this purpose, values numbered “0” were assigned the value of -7. Cox regression was used for univariate and multivariate analysis to predict the relative risk of relapse with a 95% confidence interval (CI). pLSC frequency both at diagnosis and at follow up, was defined as a percentage of total WBCs. P-values below 0.05 were considered significant.

Reference list

1. Van Rhenen A, Van Dongen GA, Kelder A, Rombouts EJ, Feller N, et al. (2007) The novel AML stem cell associated antigen CLL-1 aids in discrimination between normal and leukemic stem cells. Blood 110: 2659–2666.

2. Van Rhenen A, Feller N, Kelder A, Westra AH, Rombouts E, et al. (2005) High stem cell frequency in acute myeloid leukemia at diagnosis predicts high minimal residual disease and poor survival. Clinical cancer research 11 :6520–6527.

3. Pearce DJ, Taussig D, Zibara K, Smith LL, Ridler CM, et al. (2006) AML engraftment in the NOD/SCID assay reflects the outcome of AML: implications for our understanding of the heterogeneity of AML. Blood 107: 1166–1173.

4. Yahata T, Ando K, Sato T, Miyatake H, Nakamura Y, et al. (2003) A highly sensitive strategy for SCID-repopulating cell assay by direct injection of primitive human hematopoietic cells into NOD/SCID mice bone marrow. Blood 101: 2905–2913.
